# Observation of the Magnetic Ground State of the Two Smallest Triangular Nanographenes

**DOI:** 10.1021/jacsau.2c00666

**Published:** 2023-03-08

**Authors:** Elia Turco, Annika Bernhardt, Nils Krane, Leoš Valenta, Roman Fasel, Michal Juríček, Pascal Ruffieux

**Affiliations:** † nanotech@surfaces Laboratory, Empa−Swiss Federal Laboratories for Materials Science and Technology, 8600 Dübendorf, Switzerland; ‡ Department of Chemistry, University of Zurich, Winterthurerstrasse 190, 8057 Zurich, Switzerland; § Department of Chemistry, Biochemistry and Pharmaceutical Sciences, University of Bern, 3012 Bern, Switzerland

**Keywords:** scanning tunneling microscopy, on-surface synthesis, phenalenyl, triangulene, open-shell nanographene, Kondo effect

## Abstract

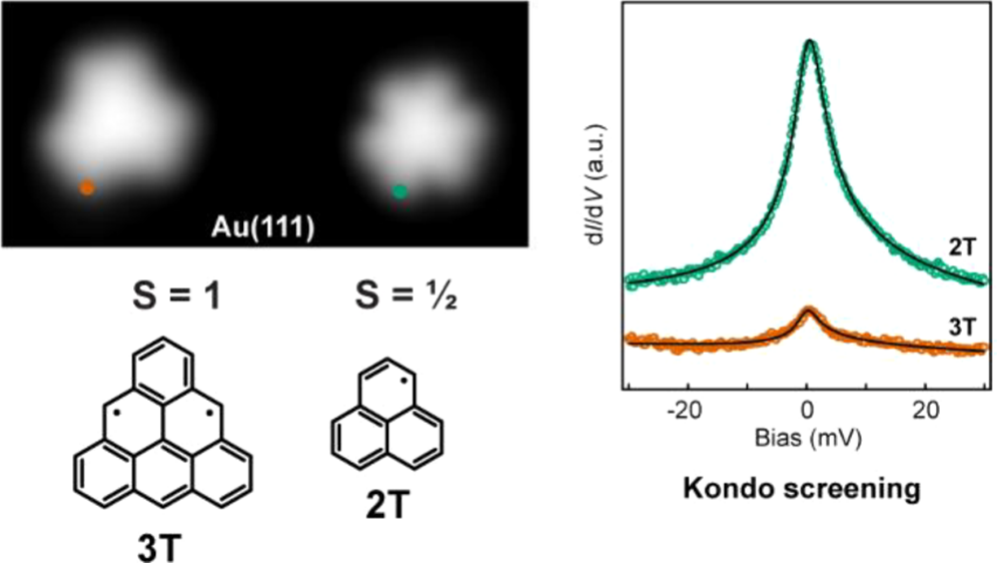

Fusion of three benzene rings in a triangular fashion gives rise to the smallest open-shell graphene fragment, the phenalenyl radical, whose π-extension leads to an entire family of non-Kekulé triangular nanographenes with high-spin ground states. Here, we report the first synthesis of unsubstituted phenalenyl on a Au(111) surface, which is achieved by combining in-solution synthesis of the hydro-precursor and on-surface activation by atomic manipulation, using the tip of a scanning tunneling microscope. Single-molecule structural and electronic characterizations confirm its open-shell *S* = 1/2 ground state that gives rise to Kondo screening on the Au(111) surface. In addition, we compare the phenalenyl’s electronic properties with those of triangulene, the second homologue in the series, whose *S* = 1 ground state induces an underscreened Kondo effect. Our results set a new lower size limit in the on-surface synthesis of magnetic nanographenes that can serve as building blocks for the realization of new exotic quantum phases of matter.

The bipartite nature of graphene’s honeycomb lattice offers the opportunity to design open-shell nanographenes (NGs) with tailor-made magnetic ground states. For specific NG topologies (also known as non-Kekulé^2^), the imbalance between the two interpenetrating lattices A and B leads to the presence of unpaired electrons forming non-trivial magnetic ground states with a total spin *S* = |*N*_A_ – *N*_B_|/2, as predicted by Ovchinnikov and Lieb about 50 years ago.^1,3^ Unlike localized *d-* or *f-*shell electrons, π-conjugated radicals are highly delocalized and prone to interact with neighboring unpaired electrons to form strongly correlated magnetic states, which are the main requisite for measurement-based quantum computation.^4^ In this regard, the family of zigzag-edged triangular NGs represent a prototypical class of polybenzenoid magnetic building blocks for the realization of such entangled spin systems. These odd-alternant triangular graphene fragments, commonly denoted as [*n*]triangulenes or simply *n*T, where *n* ≥ 2 is the number of benzene rings per edge, possess a total spin *S* that scales with triangulene size.^5^

Since the early work of Clar,^6^ the two smallest mono- and diradical polycyclic conjugated hydrocarbons 2T and 3T (Figure 1) have played a central role in the fundamental understanding of the reactivity and electronic properties of open-shell compounds. In 1957, Calvin^7^ described for the first time the phenalenyl radical (2T), formed by coincidence from phenalene by oxidation, where the presence of the radical species was confirmed by electron paramagnetic resonance spectroscopy. However, the high spin density symmetrically distributed over the majority sublattice periphery (α-positions) makes zigzag edges of 2T highly reactive and subject to σ-dimerization and oxidation in air,^8^ preventing the isolation and characterization of the pristine compound. Steric protection of the reactive sites and thermodynamic stabilization via π-extension allowed for the solution-based synthesis and characterization of persistent 2T^9−12^ and more recently 3T derivatives.^13−15^

On-surface synthesis under ultrahigh vacuum conditions turned out to be an efficient route toward the realization of unsubstituted open-shell compounds^16,17^ whose structural and electronic properties can be characterized in situ at the single-molecule level by means of scanning probe techniques. With this approach, the synthesis of unsubstituted 3T was successfully achieved on Cu(111), NaCl(100), and Xe(111) using the tip of a scanning probe microscope.^18^ Since then, the triangulene family has grown rapidly with the on-surface synthesis of even more challenging multiradical 4T,^19^ 5T,^20^ and 7T.^21^ Just recently, these magnetic building blocks were coupled into dimers,^22^ trimers,^23^ rings,^24^ and 1D chains,^25^ revealing first examples of fascinating correlated magnetic ground states.

**Figure 1:**
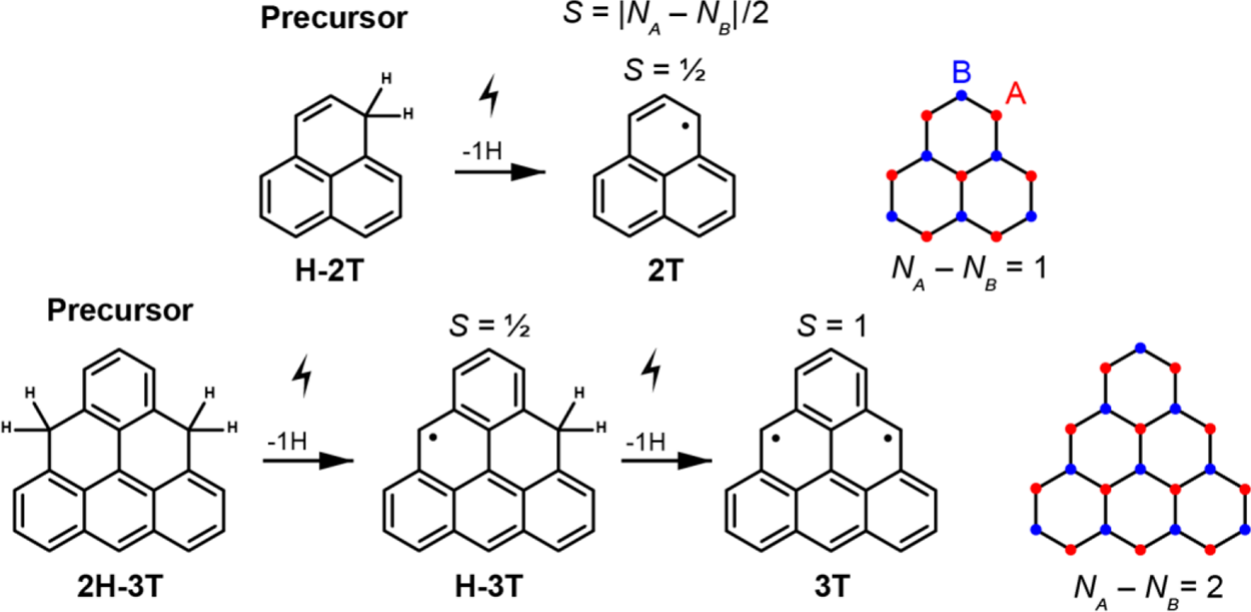
Synthesis of 2T and 3T from the corresponding hydro-precursors. Removal of one hydrogen atom from each of the *sp*^3^-hybridized carbon atoms leads to non-Kekulé polycyclic conjugated hydrocarbons 2T and 3T, where the imbalance between sublattices *N*_A_ and *N*_B_ determines the total spin quantum number *S* according to Ovchinnikov’s rule.^1^

Despite the rich in-solution and on-surface synthesis of triangular NGs, the pristine structure and electronic properties of the smallest member of the family, namely, 2T, have not yet been explored. Here, we report the synthesis of 2T and 3T following a combined in-solution and on-surface activation approach. The chosen strategy involves in-solution synthesis of non-reactive hydro-precursors, H-2T and 2H-3T, that are deposited on a metal surface under ultrahigh vacuum conditions and subsequently activated to 2T and 3T by tip-induced dehydrogenation (as depicted in Figure 1). Their in situ characterization by scanning tunneling microscopy (STM) and spectroscopy (STS) measurements yields a direct proof of the magnetic ground state of 2T and 3T on Au(111) via the observation of a Kondo resonance.

## Results and Discussion

### In-Solution Synthesis of H-2T and 2H-3T

H-2T was prepared from commercially available 3-(naphthalen-1-yl)propanoic acid in three steps. The phenalene core was built up in a Friedel–Crafts acylation. The reduction of 2,3-dihydro-1*H*-phenalen-1-one and a subsequent dehydration reaction yielded H-2T. The air-sensitive compound was purified by column chromatography and subsequent sublimation. The experimental details of the in-solution synthesis of H-2T are reported in the Supporting Information.

The synthesis of 2H-3T was achieved by combining the procedures of Šolomek and Juríček^26^ with that of Johnson.^27^ This approach makes this precursor accessible in only two steps with an overall yield of 70%. The route employs commercially available *o*-bromobenzyl alcohol that is reacted with two equivalents of *n*-butyllithium before it is quenched with diethyl carbonate to form a tetraalcohol intermediate. The cyclization of the tetraalcohol with triflic acid provides triangulenyl cation, an unstable intermediate that is reduced in situ, resulting in a mixture of two isomers of 2H-3T, as described previously.^26,27^

### On-Surface Synthesis of 2T and 3T

**Figure 2:**
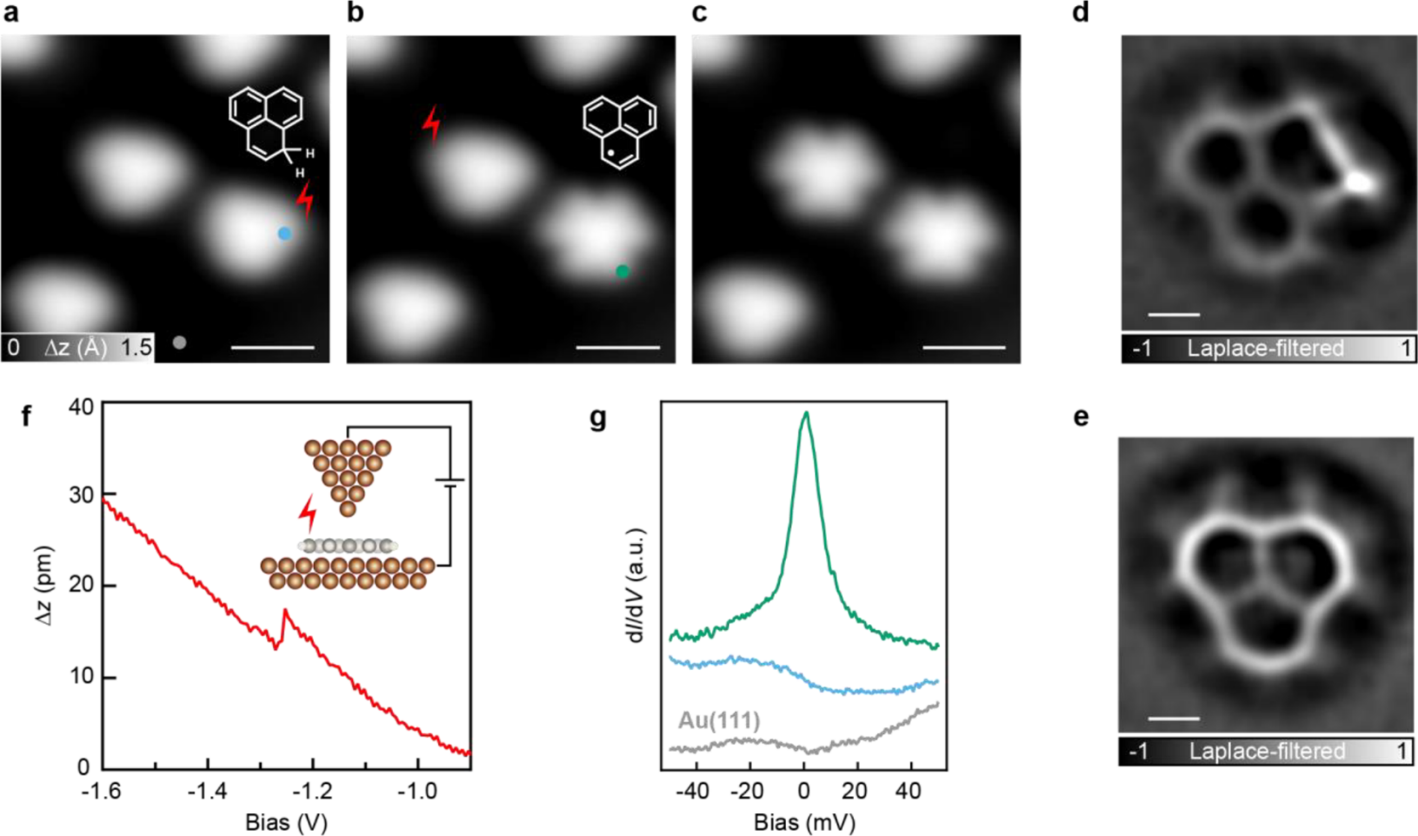
Tip-induced activation of H-2T on Au(111). (a–c) Series of STM images showing the selective dehydrogenation of H-2T to 2T (*V* = −0.6 V, *I* = 100 pA). (d, e) Laplace-filtered nc-AFM images of H-2T and 2T molecules. Open feedback parameters: *V* = −50 mV, *I* = 100 pA; *Δz* = −1.8 Å (H-2T) and −1.5 Å (2T). (f) Cleaving of the hydrogen atom is detected as a step in constant-current *z*(*V*) spectroscopy (*I* = 50 pA). (g) Constant-height d*I*/d*V* spectra acquired on H-2T and 2T, revealing the Kondo screening of the radical in 2T. Scale bars: 1 nm (a–c) and 0.2 nm (d, e).

The hydro-precursors H-2T and 2H-3T were sublimed under ultrahigh vacuum conditions onto a Au(111) surface held at room temperature. STM imaging of the resulting surface (reported in Figure S1) reveals that both precursors are adsorbed as individual molecules that are predominantly located in the face-centered cubic (fcc) regions of the herringbone reconstruction of Au(111) when deposited at submonolayer coverage. Together with the target hydro-precursors, we also observed a minority of different species, often self-assembled into molecular clusters, that we assign to oxidized molecules.^18^ Despite the presence of the additional hydrogen atom, which breaks the three-fold symmetry, STM imaging of H-2T species (Figure 2a) reveals an almost uniform triangular apparent shape, while its nonplanar chemical structure is directly resolved by bond-resolved non-contact atomic force microscopy (nc-AFM)^28^ (Figure 2d).

Dehydrogenation of H-2T and 2H-3T, i.e., the removal of one hydrogen atom from the *sp*^3^-hybridized carbon atoms, was achieved by means of atomic manipulation.^29,30^ Specifically, we positioned the STM/nc-AFM tip above one of the corners of H-2T and, while keeping the current fixed to few tens of pA, increased the voltage until a jump in tip height was observed (Figure 2f). Subsequent STM imaging (Figure 2b) revealed a significant change in the apparent shape of the target molecule, while all the other molecules remained unaffected. The successful dehydrogenation of H-2T was confirmed by nc-AFM images shown in Figure 2e, which reveal a stable and flat geometry of 2T on the Au(111) surface. In addition to STM/nc-AFM imaging, we also followed the dehydrogenation of H-2T by low-energy differential conductance (d*I*/d*V*) spectroscopy. Figure 2g shows a clear transition from a featureless low-energy conductance spectrum for the H-2T precursor to a sharp zero-bias resonance (ZBR) measured at the six equivalent lobes located at the edges of 2T. This ZBR is the characteristic fingerprint of the Kondo screening of a spin *S* = 1/2 impurity on a metal substrate, thus attesting to the open-shell ground state of the 2T species, as discussed in more detail below. We repeated the tip-based activation procedure of the precursor H-2T into 2T with different metal tips and over 50 different molecules, which revealed that dehydrogenation is bias voltage-dependent and occurs on average at a sample bias of −1.3 V. An alternative way of dehydrogenating H-2T on Au(111) consists of annealing the sample to 180 °C (see Figure S2 for details). In an analogous way to activating H-2T into 2T, triangulene 3T was generated from the dihydro-precursor 2H-3T by a tip-induced dehydrogenation of the two *sp*^3^-hybridized carbon atoms, as will be discussed later.

### Electronic Structure of 2T

**Figure 3:**
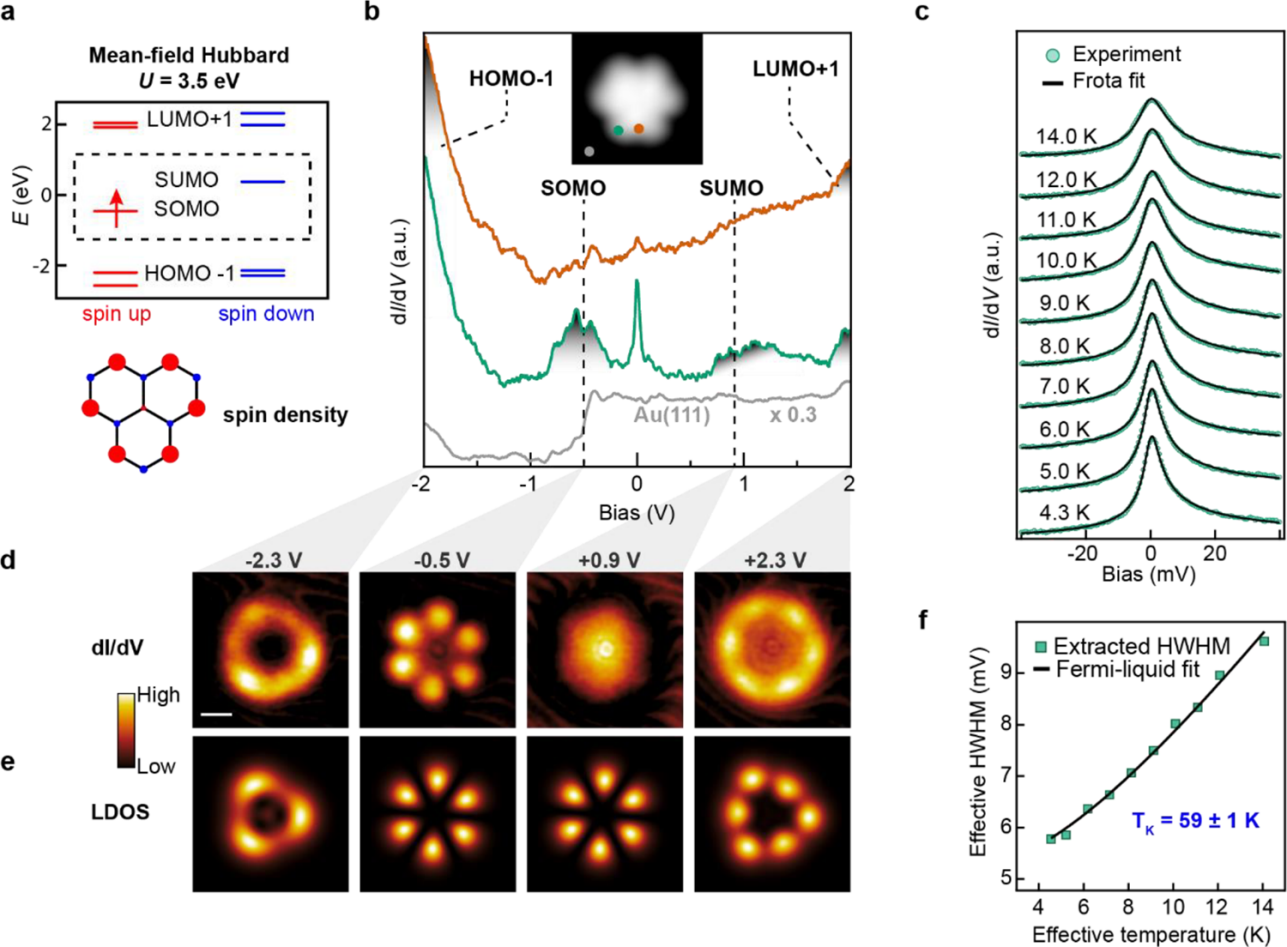
Electronic characterization of 2T. (a) MFH energy diagram of 2T, where *U* denotes the on-site Coulomb repulsion. Along with the energy spectrum, the spin density distribution is depicted, where red- and blue-filled circles denote mean populations of spin-up and spin-down electrons, respectively. (b) d*I*/d*V* spectroscopy on 2T acquired with a CO-functionalized tip reveals four molecular orbital resonances (open feedback parameters: *V* = −2.0 V, *I* = 250 pA; *V*_rms_ = 16 mV). Acquisition positions are indicated in the STM image shown in the inset. (c) Kondo resonance of 2T as a function of sample temperature (open feedback parameters: *V* = −50 mV, *I* = 1 nA; *V*_rms_ = 0.4 mV). (d, e) Constant-current d*I*/d*V* maps of the HOMO-1, SOMO, SUMO, and LUMO+1 resonances of 2T (d), along with the corresponding MFH-LDOS maps (e). Tunneling parameters for the d*I*/d*V* maps: *I* = 300 pA, *V*_rms_ = 24 mV. Scale bar: 0.5 nm. (f) Half-width at half-maximum (HWHM) of the d*I*/d*V* spectra in (c) extracted from the Frota fits and plotted versus the effective temperature, as described in ref (16). The solid black line shows the best fit with the function , which describes the thermal broadening of the Kondo resonance in the Fermi liquid model.^31^ Fitting parameters: *T*_K_ ∼ 59 K and α = 13.

The bond topology of 2T intrinsically leads to the presence of an unpaired electron, which in the tight-binding (TB) model is depicted as a non-bonding, half-filled zero-energy state. If we also consider the on-site electron–electron Coulomb repulsion *U* within the mean-field Hubbard (MFH) level of theory, this zero-energy state splits into a singly occupied and a singly unoccupied molecular orbital (SOMO/SUMO) with opposite spin orientations (Figure 3a).

**Figure 4:**
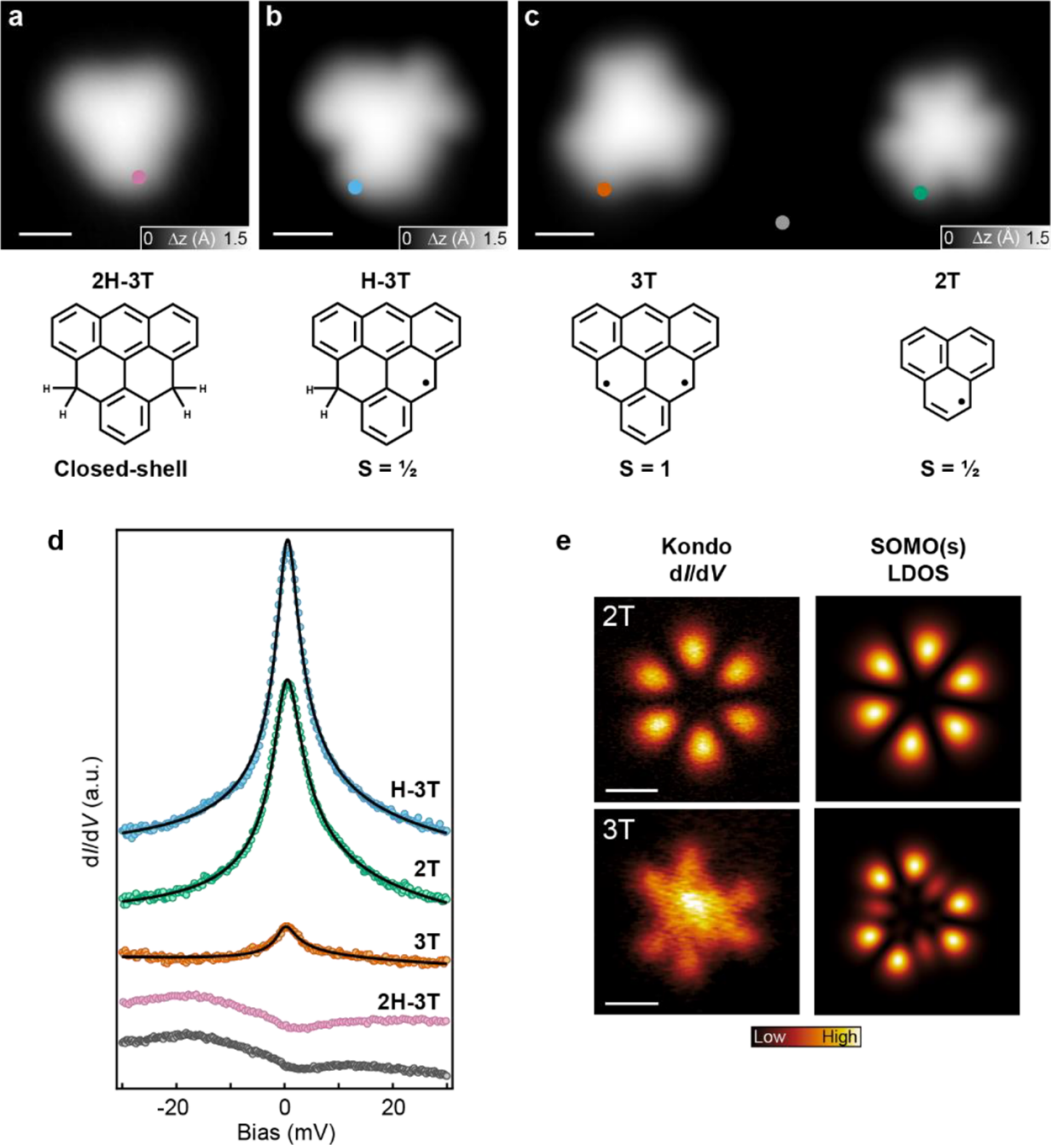
Comparison of spin-1/2 (2T) and spin-1 (3T) Kondo resonances. (a–c) High-resolution STM images of 2H-3T (*V* = −0.1 V, *I* = 20 pA), H-3T (*V* = −0.1 V, *I* = 100 pA), and 3T and 2T (*V* = −0.1 V, *I* = 100 pA) obtained by successive tip-induced dehydrogenation. (d) Low-bias d*I*/d*V* spectra (colored dots) acquired on (a–c) using a metal tip (open feedback parameters: *V* = −50 mV, *I* = 1 nA; *V*_rms_ = 0.35 mV), revealing a ZBR for the *S* = 1/2 and *S* = 1 systems. The positions of the acquired spectra are indicated in (a–c). The d*I*/d*V* background from the Au(111) substrate surface (gray curve) was subtracted from the 3T, 2T, and H-3T d*I*/d*V* spectra. Frota fits are shown as black lines (see Figure S7 for details). (e) Experimental constant-height d*I*/d*V* maps of the ZBR in 2T and 3T (left panels), with the corresponding MFH-TB LDOS SOMO(s) of 2T and 3T (right panels). A subtraction of the off-resonance d*I*/d*V* maps (*V* = 30 mV) was applied to remove the uniform d*I*/d*V* background of the molecules. Open feedback parameters; 2T: *V* = −100 mV, *I* = 1 nA; *V*_rms_ = 1 mV, 3 T: *V* = −100 mV, *I* = 1.4 nA; *V*_rms_ = 2 mV. Scale bars: 0.5 nm (a–c, e).

The unpaired electron giving rise to the net spin *S* = 1/2 of 2T is delocalized over the entire molecular framework, but with the highest probability density at the six α-positions, as shown by the spin density plot in Figure 3a. The TB-MFH theoretical predictions are entirely confirmed by our STS measurements of isolated 2T molecules on Au(111). The differential conductance d*I*/d*V* spectra, acquired at two different positions on the molecule, reveal four distinct peaks in the local density of states (LDOS), at −2.0, −0.5, +0.9, and 2.0 V (Figure 3b). To assign these resonances to the calculated MFH-LDOS, shown in Figure 3e, we did a spatial mapping of each d*I*/d*V* resonance (Figure 3d). The excellent match between the calculated LDOS and experimental d*I*/d*V* maps proves the correct assignment of the MOs. As indicated in Figure 3d,e, SOMO and SUMO share the same LDOS. Indeed, for a molecule with a singly occupied orbital, tunneling at opposite bias polarities involves adding/removing an electron to/from this orbital.^32^ The Coulomb energy penalty for doubly occupying this orbital with respect to the unoccupied case gives rise to the experimentally observed energy gap of 1.4 eV. Notably, the obtained value is comparable with the energy gap of larger triangulene homologues adsorbed on the Au(111) surface, where the well-known screening effect determines a significant reduction of the molecule’s gap if compared to *G*_0_*W*_0_ level of theory calculations^19^ or experiments on decoupling layers.^18^ Despite the identical symmetry of the experimental SOMO and SUMO d*I*/d*V* maps, the energetic overlap of the SUMO state with the gold surface state hinders its clear visualization. This can be improved by performing d*I*/d*V* mapping with a CO-terminated tip, which reveals an increased sensitivity to the SUMO (Figure S3). However, a much more direct proof for the presence of an unpaired spin on a metal surface is the detection of a ZBR in the d*I*/d*V* spectrum that is related to the Kondo screening of its magnetic moment.^33,34^ Spatial mapping of this ZBR (Figure 4e) indirectly gives access to the spin density distribution, which naturally resembles the LDOS of the singly occupied/unoccupied MOs. To confirm that the ZBR peak indeed derives from a Kondo resonance, we determined the ZBR linewidth as a function of sample temperature (Figure 3c). It is found to broaden non-thermally and to follow the characteristic trend of a *S* = 1/2 Kondo-screened state with a Kondo temperature *T*_K_ = 59 ± 1 K (Figure 3f).

### Kondo Screening in *S* = 1/2 and *S* = 1 Triangular NGs

The second point that we want to address is a direct comparison of the Kondo screening in the *S* = 1/2 and *S* = 1 NGs 2T and 3T, respectively. The first evidence of the ferromagnetic *S* = 1 ground state of 3T was reported by Pavliček et al. on non-metallic surfaces.^18^ Here, we provide direct evidence that 3T retains its triplet magnetic ground state when adsorbed on Au(111). A detailed electronic characterization of 3T is reported in Figure S5, while here we will focus on the low-energy magnetic properties. As already shown for 2T, the presence of a magnetic impurity with (2*S* + 1)-degenerate spin ground state coupled to the electron bath of the substrate results in a (complete or partial) screening of the magnetic moment of the impurity. If we have a single screening channel (the conduction band of the Au substrate), the spin of the magnetic adsorbate is screened from a spin *S* to an effective spin (*S* – 1/2).^35,36^ This implies that, for systems with *S* > 1/2, the spin is not fully screened, and one thus speaks of an underscreened Kondo effect, where the residual magnetic moment has a Zeeman energy much smaller than the Kondo temperature.^37,38^ Here, by co-depositing both H-2T and 2H-3T onto the Au(111) surface, we investigate the differences between a fully screened (*S* = 1/2, 2T) and an underscreened (*S* = 1, 3T) Kondo state. The high-resolution STM image in Figure 4a shows the typical apparent shape of 2H-3T, where the two additional hydrogen atoms (one at each *sp*^3^ center) ″quench″ the diradical nature, resulting in a featureless low-energy d*I*/d*V* spectrum (Figure 4d). Employing the aforementioned manipulation technique, we first selectively removed one hydrogen atom, inducing a clear modification in the apparent shape of the molecule (Figure 4b). The so obtained H-3T molecule reveals a strong ZBR characteristic of a Kondo-screened *S* = 1/2 ground state. We then proceeded and removed the second hydrogen atom, to obtain the target compound 3T (Figure 4c, left) featuring a three-fold symmetric apparent shape. In order to compare the Kondo-related ZBR of 2T and 3T with the same metal tip, we also activated a nearby 2T molecule (Figure 4c, right) and performed low-bias STS on both 2T and 3T.

The low-energy differential conductance spectra shown in Figure 4d reveal a pronounced intensity difference of the ZBRs of 2T and 3T. From a Frota fit^39,40^ to the 2T and 3T spectra, we determined an amplitude ratio of 7 to 1. This remarkable difference is consistent with recent observations on similar *S* = 1/2 and *S* = 1 systems,^41−43^ where significantly reduced intensity for underscreened Kondo resonances was reported. According to early experiments on *d*-electron materials^44^ and more recent renormalization group studies within the Anderson model,^45^ the Hund coupling of the spins in a *S* > 1/2 ground state determines a quenching of the effective Kondo coupling, which results in an exponentially reduced Kondo temperature compared to a *S* = 1/2 Kondo system. Considering the strong Hund coupling of hundreds of meV in 3T^46,47^ and the recent experimental results on similar *S* = 1 NGs, we expect a Kondo temperature within our limited experimentally accessible temperature range (4.5 K < *T* < 14 K). Therefore, a correct estimation of the Kondo temperature would require a wider range in temperatures and a tunable magnetic field,^48,49^ which is beyond our current experimental capabilities. Nevertheless, since the ZBRs of 2T and 3T were measured in the very same conditions, we expect that a lower Kondo temperature would correspond to a narrower HWHM. Frota fitting of 2T and 3T spectra revealed that the ZBR of 3T is significantly narrower than 2T ZBR, supporting the aforementioned hypothesis of a lower Kondo temperature of 3T. More details on the HWHMs of each ZBR are reported in Figure S7.

In summary, we have reported the synthesis of unsubstituted [2]triangulene (phenalenyl radical, 2T) and [3]triangulene (3T) on Au(111) via tip-induced dehydrogenation of hydro-/dihydro-precursors, respectively. Single-molecule STM/nc-AFM and STS measurements have provided a comprehensive characterization of their chemical structure as well as their electronic and magnetic properties. A direct proof for their open-shell *S* = 1/2 and *S* = 1 ground state, respectively, has been obtained by the observation of Kondo screening of the unpaired spin(s) by the Au substrate electrons. Notably, the ferromagnetically coupled spins in triangulene 3T give rise to an underscreened Kondo effect, as recently predicted by multiorbital Anderson impurity model calculations.^50^ On the other hand, the phenalenyl radical 2T with its three-fold symmetry and spin-1/2 doublet ground state constitutes a prototypical all-carbon magnetic building block, whose successful on-surface synthesis and characterization opens new opportunities for the bottom-up synthesis of strongly correlated one- and two-dimensional carbon-based spin chains and lattices.

## Supporting Information Available

Experimental and computational methods, supporting STM and STS data, additional calculations, Frota fitting parameters, and a detailed synthetic description of chemical compounds reported in this study (PDF)

## References

[ref2] Randić M. Aromaticity of Polycyclic Conjugated Hydrocarbons. Chem. Rev. 2003, 103, 3449–3606. 10.1021/cr9903656. 12964878

[ref1] Ovchinnikov A. A. Multiplicity of the Ground State of Large Alternant Organic Molecules with Conjugated Bonds. Theor. Chim. Acta 1978, 47, 297–304. 10.1007/BF00549259.

[ref3] Lieb E. H. Two Theorems on the Hubbard Model. Phys. Rev. Lett. 1989, 62, 1201–1204. 10.1103/PhysRevLett.62.1201. 10039602

[ref4] Briegel H. J.; Browne D. E.; Dür W.; Raussendorf R.; Van den Nest M. Measurement-Based Quantum Computation. Nat. Phys. 2009, 5, 19–26. 10.1038/nphys1157.

[ref5] Su J.; Telychko M.; Song S.; Lu J. Triangulenes: From Precursor Design to On-Surface Synthesis and Characterization. Angew. Chem., Int. Ed. 2020, 59, 7658–7668. 10.1002/anie.201913783. 31872494

[ref6] Clar E.; Stewart D. G. Aromatic Hydrocarbons. LXV. Triangulene Derivatives. J. Am. Chem. Soc. 1953, 75, 2667–2672. 10.1021/ja01107a035.

[ref7] Sogo P. B.; Nakazaki M.; Calvin M. Free Radical from Perinaphthene. J. Chem. Phys. 1957, 26, 1343–1345. 10.1063/1.1743526.

[ref8] Uchida K.; Kubo T. Recent Advances in the Chemistry of Phenalenyl. J. Synth. Org. Chem., Jpn. 2016, 74, 1069–1077. 10.5059/yukigoseikyokaishi.74.1069.

[ref9] Goto K.; Kubo T.; Yamamoto K.; Nakasuji K.; Sato K.; Shiomi D.; Takui T.; Kubota M.; Kobayashi T.; Yakusi K.; Ouyang J. A Stable Neutral Hydrocarbon Radical: Synthesis, Crystal Structure, and Physical Properties of 2,5,8-Tri-Tert-Butyl-Phenalenyl. J. Am. Chem. Soc. 1999, 121, 1619–1620. 10.1021/ja9836242.

[ref10] Kubo T. Phenalenyl-Based Open-Shell Polycyclic Aromatic Hydrocarbons. Chem. Rec. 2015, 15, 218–232. 10.1002/tcr.201402065. 25345729

[ref11] Yang Y.; Blacque O.; Sato S.; Juríček M. Cycloparaphenylene–Phenalenyl Radical and Its Dimeric Double Nanohoop. Angew. Chem., Int. Ed. 2021, 60, 13529–13535. 10.1002/anie.202101792. PMC8252656 33635576

[ref12] Mou Z.; Uchida K.; Kubo T.; Kertesz M. Evidence of σ- and π-Dimerization in a Series of Phenalenyls. J. Am. Chem. Soc. 2014, 136, 18009–18022. 10.1021/ja509243p. 25394519

[ref13] Arikawa S.; Shimizu A.; Shiomi D.; Sato K.; Shintani R. Synthesis and Isolation of a Kinetically Stabilized Crystalline Triangulene. J. Am. Chem. Soc. 2021, 143, 19599–19605. 10.1021/jacs.1c10151. 34767718

[ref14] Valenta L.; Mayländer M.; Kappeler P.; Blacque O.; Šolomek T.; Richert S.; Juríček M. Trimesityltriangulene: A Persistent Derivative of Clar’s Hydrocarbon. Chem. Commun. 2022, 58, 3019–3022. 10.1039/D2CC00352J. PMC8886921 35156113

[ref15] Valenta L.; Juríček M. The Taming of Clar’s Hydrocarbon. Chem. Commun. 2022, 58, 10896–10906. 10.1039/D2CC03720C. PMC9521324 36098074

[ref16] Mishra S.; Beyer D.; Eimre K.; Kezilebieke S.; Berger R.; Gröning O.; Pignedoli C. A.; Müllen K.; Liljeroth P.; Ruffieux P.; Feng X.; Fasel R. Topological Frustration Induces Unconventional Magnetism in a Nanographene. Nat. Nanotechnol. 2020, 15, 22–28. 10.1038/s41565-019-0577-9. 31819244

[ref17] Turco E.; Mishra S.; Melidonie J.; Eimre K.; Obermann S.; Pignedoli C. A.; Fasel R.; Feng X.; Ruffieux P. On-Surface Synthesis and Characterization of Super-Nonazethrene. J. Phys. Chem. Lett. 2021, 12, 8314–8319. 10.1021/acs.jpclett.1c02381. 34428064

[ref18] Pavliček N.; Mistry A.; Majzik Z.; Moll N.; Meyer G.; Fox D. J.; Gross L. Synthesis and Characterization of Triangulene. Nat. Nanotechnol. 2017, 12, 308–311. 10.1038/nnano.2016.305. 28192389

[ref19] Mishra S.; Beyer D.; Eimre K.; Liu J.; Berger R.; Gröning O.; Pignedoli C. A.; Müllen K.; Fasel R.; Feng X.; Ruffieux P. Synthesis and Characterization of π-Extended Triangulene. J. Am. Chem. Soc. 2019, 141, 10621–10625. 10.1021/jacs.9b05319. 31241927

[ref20] Su J.; Telychko M.; Hu P.; Macam G.; Mutombo P.; Zhang H.; Bao Y.; Cheng F.; Huang Z.-Q.; Qiu Z.; Tan S. J. R.; Lin H.; Jelínek P.; Chuang F.-C.; Wu J.; Lu J. Atomically Precise Bottom-up Synthesis of π-Extended [5]Triangulene. Sci. Adv. 2019, 5, eaav7717 10.1126/sciadv.aav7717. 31360763 PMC6660211

[ref21] Mishra S.; Xu K.; Eimre K.; Komber H.; Ma J.; Pignedoli C. A.; Fasel R.; Feng X.; Ruffieux P. Synthesis and Characterization of [7]Triangulene. Nanoscale 2021, 13, 1624–1628. 10.1039/D0NR08181G. 33443270

[ref22] Mishra S.; Beyer D.; Eimre K.; Ortiz R.; Fernández-Rossier J.; Berger R.; Gröning O.; Pignedoli C. A.; Fasel R.; Feng X.; Ruffieux P. Collective All-Carbon Magnetism in Triangulene Dimers. Angew. Chem., Int. Ed. 2020, 59, 12041–12047. 10.1002/anie.202002687. PMC7383983 32301570

[ref23] Cheng S.; Xue Z.; Li C.; Liu Y.; Xiang L.; Ke Y.; Yan K.; Wang S.; Yu P. On-Surface Synthesis of Triangulene Trimers via Dehydration Reaction. Nat. Commun. 2022, 13, 1705 10.1038/s41467-022-29371-9. 35361812 PMC8971457

[ref24] Hieulle J.; Castro S.; Friedrich N.; Vegliante A.; Lara F. R.; Sanz S.; Rey D.; Corso M.; Frederiksen T.; Pascual J. I.; Peña D. On-Surface Synthesis and Collective Spin Excitations of a Triangulene-Based Nanostar. Angew. Chem., Int. Ed. 2021, 60, 25224–25229. 10.1002/anie.202108301. PMC9292598 34647398

[ref25] Mishra S.; Catarina G.; Wu F.; Ortiz R.; Jacob D.; Eimre K.; Ma J.; Pignedoli C. A.; Feng X.; Ruffieux P.; Fernández-Rossier J.; Fasel R. Observation of Fractional Edge Excitations in Nanographene Spin Chains. Nature 2021, 598, 287–292. 10.1038/s41586-021-03842-3. 34645998

[ref26] Ribar P.; Šolomek T.; Juríček M. Gram-Scale Synthesis and Supramolecular Complex of Precursors of Clar’s Hydrocarbon Triangulene. Org. Lett. 2019, 21, 7124–7128. 10.1021/acs.orglett.9b02683. 31414815 PMC6737831

[ref27] Holt C. J.; Wentworth K. J.; Johnson R. P. A Short and Efficient Synthesis of the [3]Triangulene Ring System. Angew. Chem., Int. Ed. 2019, 58, 15793–15796. 10.1002/anie.201907226. 31489748

[ref28] Gross L.; Mohn F.; Moll N.; Liljeroth P.; Meyer G. The Chemical Structure of a Molecule Resolved by Atomic Force Microscopy. Science 2009, 325, 1110 10.1126/science.1176210. 19713523

[ref29] Schuler B.; Fatayer S.; Mohn F.; Moll N.; Pavliček N.; Meyer G.; Peña D.; Gross L. Reversible Bergman Cyclization by Atomic Manipulation. Nat. Chem. 2016, 8, 220–224. 10.1038/nchem.2438. 26892552

[ref30] Pavliček N.; Schuler B.; Collazos S.; Moll N.; Pérez D.; Guitián E.; Meyer G.; Peña D.; Gross L. On-Surface Generation and Imaging of Arynes by Atomic Force Microscopy. Nat. Chem. 2015, 7, 623–628. 10.1038/nchem.2300. 26201737

[ref31] Nagaoka K.; Jamneala T.; Grobis M.; Crommie M. F. Temperature Dependence of a Single Kondo Impurity. Phys. Rev. Lett. 2002, 88, 077205 10.1103/PhysRevLett.88.077205. 11863936

[ref32] Repp J.; Meyer G.; Paavilainen S.; Olsson F. E.; Persson M. Imaging Bond Formation Between a Gold Atom and Pentacene on an Insulating Surface. Science 2006, 312, 1196–1199. 10.1126/science.1126073. 16728636

[ref33] Kondo J. Resistance Minimum in Dilute Magnetic Alloys. Prog. Theor. Phys. 1964, 32, 37–49. 10.1143/PTP.32.37.

[ref34] Ternes M.; Heinrich A. J.; Schneider W.-D. Spectroscopic Manifestations of the Kondo Effect on Single Adatoms. J. Phys. Condens. Matter 2009, 21, 053001 10.1088/0953-8984/21/5/053001. 21817287

[ref35] Mattis D. C. Symmetry of Ground State in a Dilute Magnetic Metal Alloy. Phys. Rev. Lett. 1967, 19, 1478–1481. 10.1103/PhysRevLett.19.1478.

[ref36] Coleman P.; Pépin C. Singular Fermi Liquid Behavior in the Underscreened Kondo Model. Phys. Rev. B 2003, 68, 220405 10.1103/PhysRevB.68.220405.

[ref37] Roch N.; Florens S.; Costi T. A.; Wernsdorfer W.; Balestro F. Observation of the Underscreened Kondo Effect in a Molecular Transistor. Phys. Rev. Lett. 2009, 103, 197202 10.1103/PhysRevLett.103.197202. 20365950

[ref38] Sasaki S.; De Franceschi S.; Elzerman J. M.; van der Wiel W. G.; Eto M.; Tarucha S.; Kouwenhoven L. P. Kondo Effect in an Integer-Spin Quantum Dot. Nature 2000, 405, 764–767. 10.1038/35015509. 10866190

[ref39] Gruber M.; Weismann A.; Berndt R. The Kondo Resonance Line Shape in Scanning Tunnelling Spectroscopy: Instrumental Aspects. J. Phys. Condens. Matter 2018, 30, 424001 10.1088/1361-648X/aadfa3. 30191885

[ref40] Frota H. O. Shape of the Kondo Resonance. Phys. Rev. B 1992, 45, 1096–1099. 10.1103/PhysRevB.45.1096. 10001582

[ref41] Wang T.; Berdonces-Layunta A.; Friedrich N.; Vilas-Varela M.; Calupitan J. P.; Pascual J. I.; Peña D.; Casanova D.; Corso M.; de Oteyza D. G. Aza-Triangulene: On-Surface Synthesis and Electronic and Magnetic Properties. J. Am. Chem. Soc. 2022, 144, 4522–4529. 10.1021/jacs.1c12618. 35254059 PMC8931755

[ref42] Li J.; Sanz S.; Castro-Esteban J.; Vilas-Varela M.; Friedrich N.; Frederiksen T.; Peña D.; Pascual J. I. Uncovering the Triplet Ground State of Triangular Graphene Nanoflakes Engineered with Atomic Precision on a Metal Surface. Phys. Rev. Lett. 2020, 124, 177201 10.1103/PhysRevLett.124.177201. 32412280

[ref43] Su X.; Li C.; Du Q.; Tao K.; Wang S.; Yu P. Atomically Precise Synthesis and Characterization of Heptauthrene with Triplet Ground State. Nano Lett. 2020, 20, 6859–6864. 10.1021/acs.nanolett.0c02939. 32787160

[ref44] Daybell M. D.; Steyert W. A. Localized Magnetic Impurity States In Metals: Some Experimental Relationships. Rev. Mod. Phys. 1968, 40, 380–389. 10.1103/RevModPhys.40.380.

[ref45] Nevidomskyy A. H.; Coleman P. Kondo Resonance Narrowing in d- and f-Electron Systems. Phys. Rev. Lett. 2009, 103, 147205 10.1103/PhysRevLett.103.147205. 19905601

[ref46] Das A.; Müller T.; Plasser F.; Lischka H. Polyradical Character of Triangular Non-Kekulé Structures, Zethrenes, p-Quinodimethane-Linked Bisphenalenyl, and the Clar Goblet in Comparison: An Extended Multireference Study. J. Phys. Chem. A 2016, 120, 1625–1636. 10.1021/acs.jpca.5b12393. 26859789 PMC4789636

[ref47] Ortiz R.; Boto R. A.; García-Martínez N.; Sancho-García J. C.; Melle-Franco M.; Fernández-Rossier J. Exchange Rules for Diradical π-Conjugated Hydrocarbons. Nano Lett. 2019, 19, 5991–5997. 10.1021/acs.nanolett.9b01773. 31365266

[ref48] Zhang Y.; Kahle S.; Herden T.; Stroh C.; Mayor M.; Schlickum U.; Ternes M.; Wahl P.; Kern K. Temperature and Magnetic Field Dependence of a Kondo System in the Weak Coupling Regime. Nat. Commun. 2013, 4, 2110 10.1038/ncomms3110. 23817525 PMC3730050

[ref49] Žonda M.; Stetsovych O.; Korytár R.; Ternes M.; Temirov R.; Raccanelli A.; Tautz F. S.; Jelínek P.; Novotný T.; Švec M. Resolving Ambiguity of the Kondo Temperature Determination in Mechanically Tunable Single-Molecule Kondo Systems. J. Phys. Chem. Lett. 2021, 12, 6320–6325. 10.1021/acs.jpclett.1c01544. 34228474

[ref50] Jacob D.; Ortiz R.; Fernández-Rossier J. Renormalization of Spin Excitations and Kondo Effect in Open-Shell Nanographenes. Phys. Rev. B 2021, 104, 075404 10.1103/PhysRevB.104.075404.

