# Supplementary material for: Observation of the Magnetic Ground State of the Two Smallest Triangular Nanographenes
Source: JACS Au. 2023 Mar 8;3(5):1358–64. doi: 10.1021/jacsau.2c00666 (PMC10207087; doi:10.1021/jacsau.2c00666)
Supplement: Supplementary file 1 — au2c00666_si_001.pdf [file au2c00666_si_001.pdf]

Supporting Information

Observation of the magnetic ground state of the two smallest triangular nanographenes

Elia Turco,[⊥] Annika Bernhardt,[||] Nils Krane,[⊥] Leoš Valenta,[||] Roman Fasel,[⊥,§] Michal Juríček,[||,] and Pascal Ruffieux[⊥,*]*

⊥ nanotech@surfaces Laboratory, Empa − Swiss Federal Laboratories for Materials Science and Technology, 8600 Dübendorf, Switzerland

|| Department of Chemistry, University of Zurich, Winterthurerstrasse 190, 8057 Zurich, Switzerland

§ Department of Chemistry, Biochemistry and Pharmaceutical Sciences, University of Bern, 3012 Bern, Switzerland

* Corresponding Authors:

michal.juricek@chem.uzh.ch

pascal.ruffieux@empa.ch

Contents

1. Methods
2. Supporting STM, STS and theoretical data
3. Synthetic procedures
4. High-resolution mass spectra and NMR characterization
6. References

1. Methods

Sample Preparation and Scanning Probe Measurements

STM measurements were performed with a commercial low-temperature STM/AFM from Scienta Omicron operated at a temperature of 4.5 K and base pressure below 5×10^{-11} mbar. The Au(111) single crystal surfaces were prepared by iterative Ar^+ sputtering and annealing cycles. Prior to sublimation of molecules, the surface quality was checked through STM imaging. The powders of H-2T and 2H-3T precursors were filled into quartz crucibles of a home-built evaporator and sublimed at 70 °C and 130 °C, respectively, onto the single crystal surfaces held at room temperature. STM images were acquired both in constant-current (overview and high-resolution imaging) and constant-height (bond-resolved imaging) modes, d*I*/d*V* spectra were acquired in constant-height mode and d*I*/d*V* maps were acquired in constant-current mode. Indicated bias voltages are given with respect to the sample. Unless otherwise noted, all measurements were performed with metallic tips. Differential conductance d*I*/d*V* spectra and maps were obtained with a lock-in amplifier. Modulation voltages (root mean square amplitude V_{rms}) for each measurement are provided in the respective figure caption. Bond-resolved nc-AFM (STM) images were acquired in constant-height mode with CO-functionalized tips at low bias voltages while recording frequency and current signals. Open feedback parameters on the molecular species and subsequent lowering of the tip height (Δz) for each image are provided in the respective figure captions. The data was processed with Wavemetrics Igor Pro software[1].

Tight-binding and mean-field Hubbard calculations

TB-MFH calculations were performed by numerically solving the mean-field Hubbard Hamiltonian with third-nearest-neighbor hopping

$$\hat{H}_{MFH} = \sum_{j} \sum_{\langle \alpha,\beta \rangle_{j},\sigma} -t_j c_{\alpha,\sigma}^{\dagger} c_{\beta,\sigma} + U \sum_{\alpha,\sigma} \langle n_{\alpha,\sigma} \rangle n_{\alpha,\bar{\sigma}} - U \sum_{\alpha} \langle n_{\alpha,\uparrow} \rangle \langle n_{\alpha,\downarrow} \rangle. \quad \text{(S1)}$$

Here, $c_{\alpha,\sigma}^{\dagger}$ and $c_{\beta,\sigma}$ denote the spin selective ($\sigma \in \{\uparrow, \downarrow\}$ with $\bar{\sigma} \in \{\downarrow, \uparrow\}$) creation and annihilation operator at sites α and β, $\langle \alpha, \beta \rangle_j$ ($j = \{1, 2, 3\}$) denotes the nearest-neighbor, second-nearest-neighbor and third-nearest-neighbor sites for j = 1, 2 and 3, respectively, t_j denotes the corresponding hopping parameters (with t_1 = 2.7 eV, t_2 = 0.1 eV and t_3 = 0.27 eV for nearest-neighbor, second-nearest-neighbor and third-nearest-neighbor hopping[3]), U denotes the on-site Coulomb repulsion, $n_{\alpha,\sigma}$ denotes the number operator, and $\langle n_{\alpha,\sigma} \rangle$ denotes the mean occupation number at site α. Orbital electron densities, ρ, of the nth-eigenstate with energy E_n have been simulated from the corresponding state vector $a_{n,i,\sigma}$ by

$$\rho_{n,\sigma}(\vec{r}) = \left| \sum_{i} a_{n,i,\sigma} \phi_{2p_z}(\vec{r} - \vec{r}_i) \right|^2, \quad \text{(S2)}$$

where i denotes the atomic site index and ϕ_{2p_z} denotes the Slater $2p_z$ orbital for carbon.

All the TB-MFH calculations presented in the manuscript were done in the third-nearest-neighbor approximation and using an on-site Coulomb term U = 3.5 eV.

The TB-MFH software library[4] was developed within the Python programming language. The code is open-source, available at https://github.com/eimrek/tb-mean-field-hubbard.

2. Supporting STM, STS and theoretical data

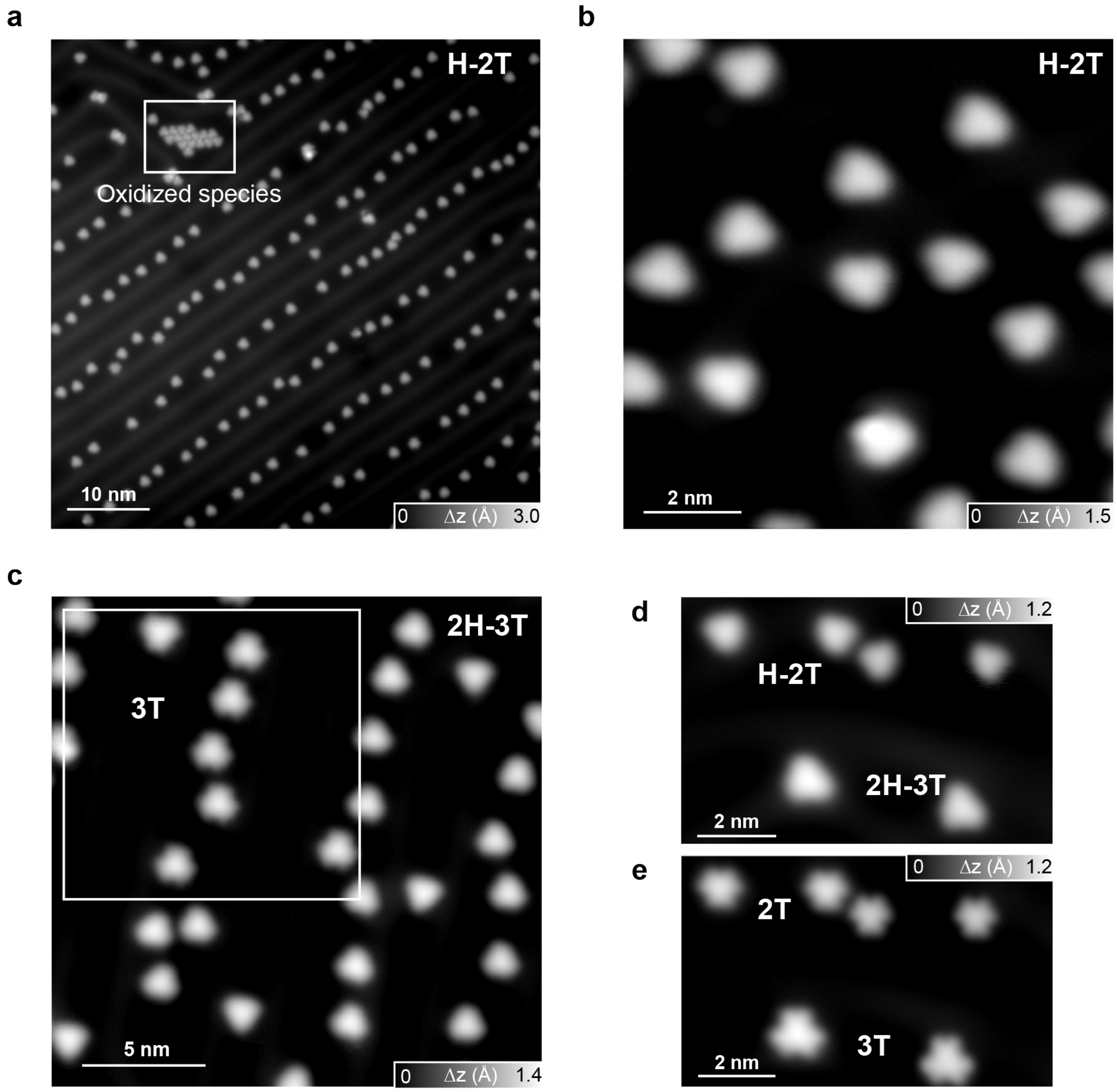

Figure S1. (**a**,**b**) Overview STM image of sub-monolayer coverage of H-2T as deposited on a Au(111) surface held at room temperature. The inset in (a) highlights the presence of self-assembled structures that we assign to oxidized species. (**c**) Overview STM image of sub-monolayer coverage of 2H-3T as deposited on a Au(111) surface at room temperature. The 3T molecules highlighted in the white frame were formerly activated by tip-induced dehydrogenation of 2H-3T precursors. (**d,e**) Sample with both H-2T and 2H-3T precursors, as deposited onto the Au(111) surface (d), and after the tip-based activation of all the molecular species (e). Scanning parameters: (a) $V = -1$ V, $I = 50$ pA; (b) $V = -0.8$ V, $I = 200$ pA; (c) $V = -0.6$ V, $I = 100$ pA; (d,e) $V = -1$ V, $I = 50$ pA.

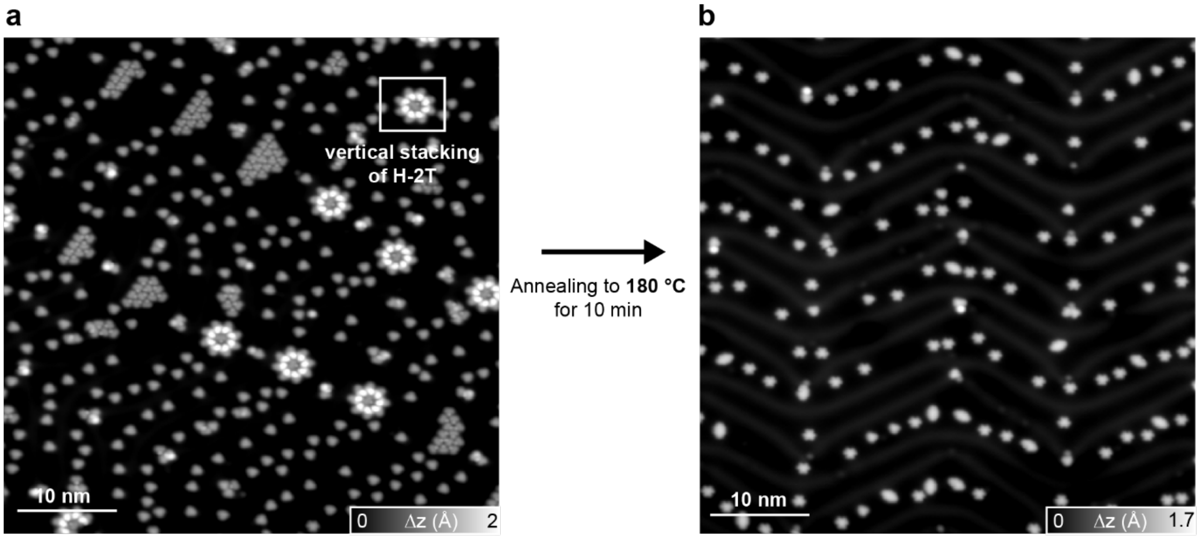

Figure S2. (**a**) Overview STM images of high-coverage sample of H-2T. (**b**) Thermally induced dehydrogenation of H-2T into the target 2T by annealing the Au(111) surface at 180 °C for 10 min. Notably, the annealing step reduces the surface coverage substantially due to monomer desorption. From the remaining molecules, ca. 70% of H-2T are converted to 2T. A minority of 2T has fused into structures with apparent rhombic shape, which we assign to peropyrenes. Scanning parameters (a,b): $V = –0.6$ V, $I = 100$ pA.

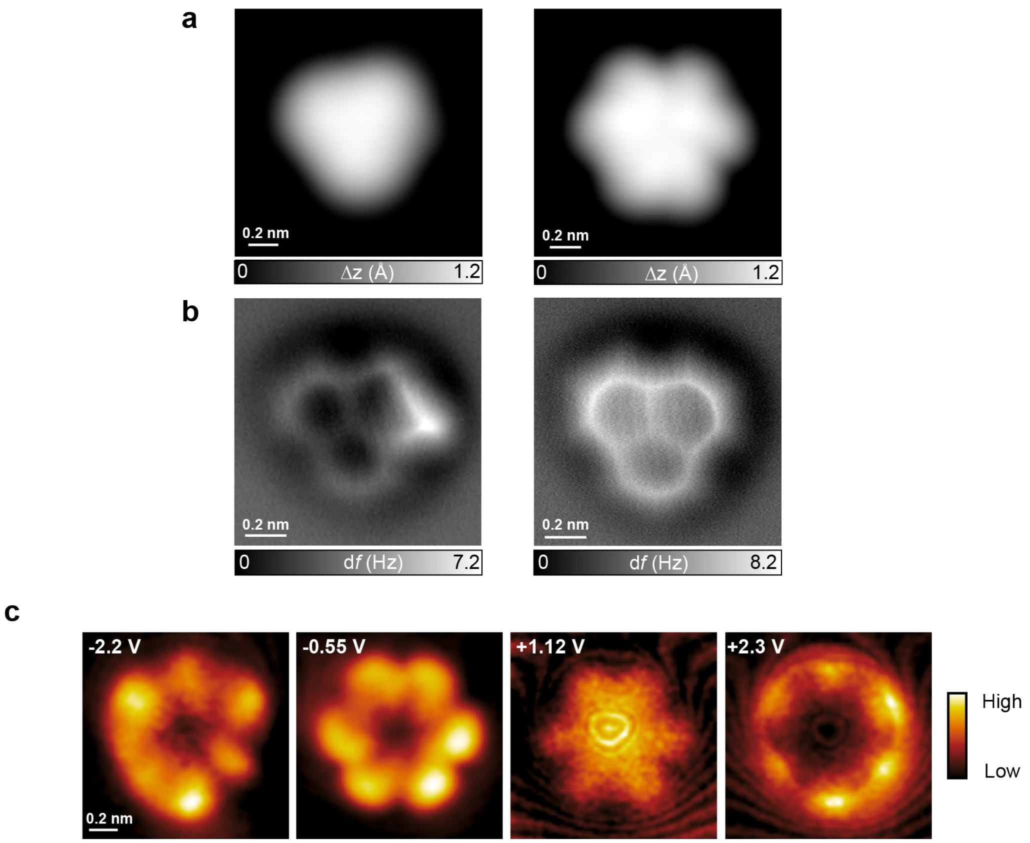

Figure S3. (**a**) High-resolution STM images for H-2T (left) and 2T (right) acquired with a CO-functionalized tip ($V = -100$ mV, $I = 100$ pA). (**b**) Corresponding nc-AFM image of H-2T and 2T (from left to right). Open feedback parameters: $V = -50$ mV, $I = 100$ pA; $\Delta z = -1.8$ Å (H-2T) and -1.5 Å (2T). (**c**) Constant-current dI/dV maps of the molecular orbital resonances, acquired with a CO-functionalized tip. All the dI/dV maps were acquired with a lock-in modulation $V_{rms} = 16$ mV and a current setpoint $I = 300$ pA.

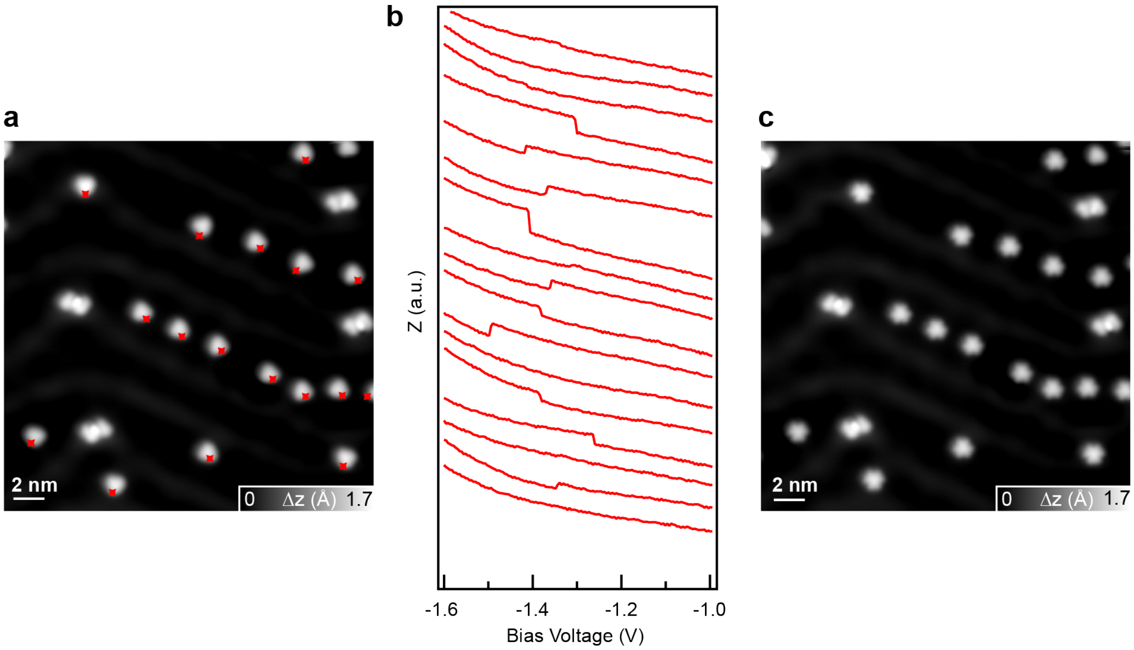

Figure S4. (**a**,**b**) Statistics on the tip-induced activation of H-2T molecules by $z(V)$ spectroscopy. (**a**) STM image of H-2T on Au(111) ($V = -100$ mV, $I = 100$ pA) where the $z(V)$ spectroscopy positions are depicted by red markers. (**b**) Cleaving of the hydrogen is detected as a step in $z(V)$ spectroscopy ($I = 50$ pA). Some spectra do not show a clear step. However, STM imaging (c) after the series of $z(V)$ spectra demonstrates the successful dehydrogenation of all targeted H-2T molecules into 2T ($V = -100$ mV, $I = 100$ pA).

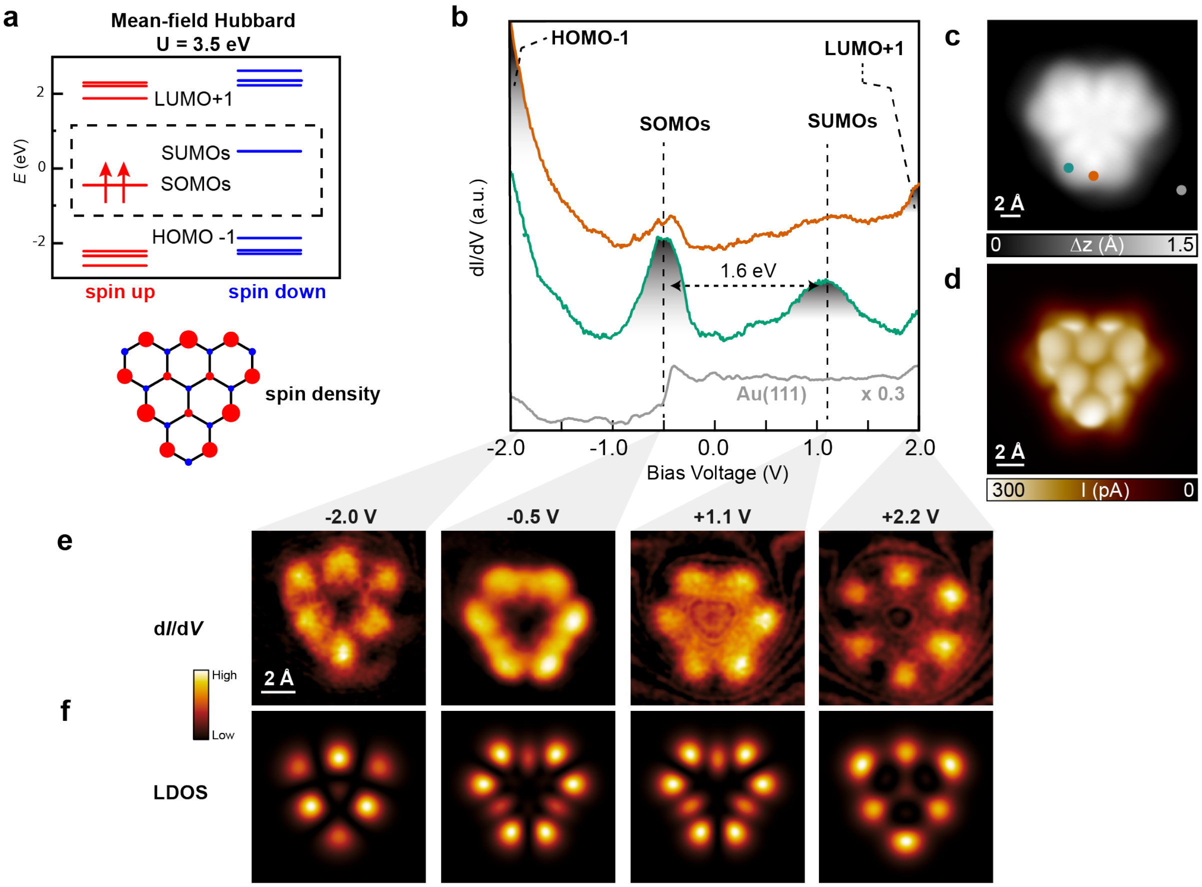

Figure S5. Electronic characterization of 3T. (**a**) MFH energy spectrum of 3T and spin polarization plot, where blue and red filled circles denote mean populations of spin up and spin down electrons, respectively. (**b**) dI/dV spectroscopy on 3T revealing molecular orbital resonances (open feedback parameters: V = –2.0 V, I = 300 pA; V_{rms} = 16 mV). Acquisition positions are indicated in the HR-STM image shown in (c) (V = –2.0 V, I = 100 pA). (**d**) Bond-resolved STM image (V = –5 mV, I = 50 pA, Δh = –0.75 Å) of 3T on Au(111), acquired with a carbon monoxide (CO) functionalized tip. (**e**) Constant-current dI/dV maps of the molecular orbital resonances, acquired with a CO-functionalized tip. All the dI/dV maps were acquired with a lock-in modulation V_{rms} = 16 mV and a current setpoint I = 200 pA. (**f**) MFH-TB LDOS of HOMO-1, SOMOs, SUMOs, LUMO+1.

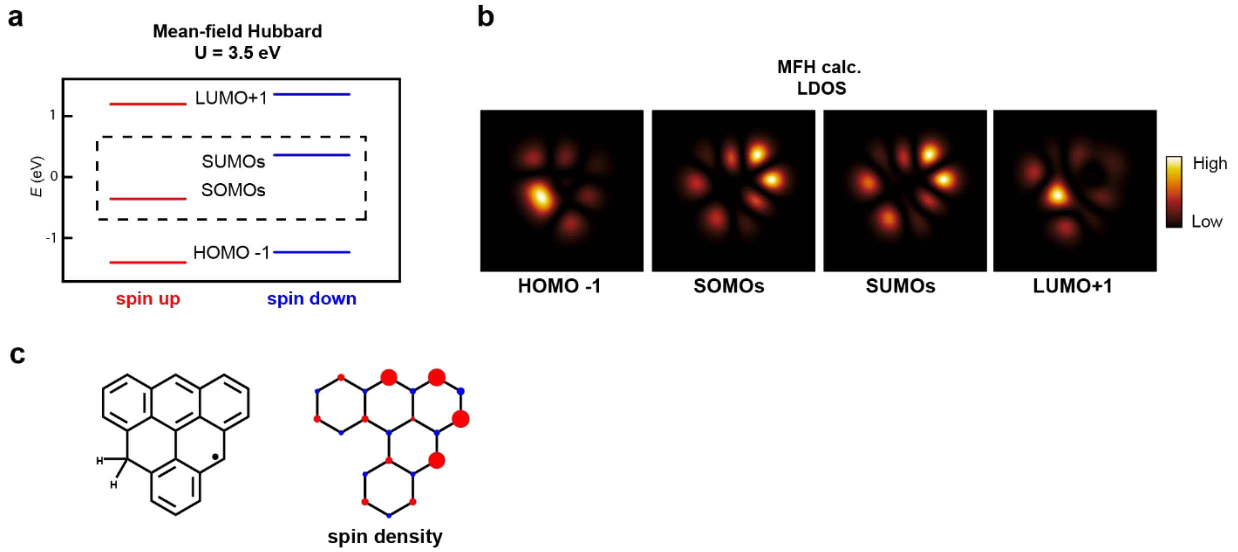

Figure S6 MFH-TB calculations of H-3T. (**a**) MFH energy spectrum of H-3T and spin polarization plot, where blue and red filled circles denote mean populations of spin up and spin down electrons, respectively. The presence of the sp^3-hybridized carbon is taken in account by removing the respective carbon atom in the MFH-TB calculation. (**b**) MFH-TB LDOS of HOMO-1, SOMO, SUMO and LUMO+1 resonances of H-3T. The frontier states LDOS matches well with the apparent shape of the HR-STM image (Fig. 4b).

Frota fit	2T	H-3T	3T
Γ (meV)	6.1 ± 0.1	4.7 ± 0.1	4.3 ± 0.3
x_0 (meV)	(3.0 ± 0.2)·10^{-5}	(4.6 ± 0.3)·10^{-4}	(0.27 ± 1.1)·10^{-5}
a	0.87 ± 0.01	1.11 ± 0.05	0.23 ± 0.01
b	-0.90 ± 0.1	-0.66 ± 0.01	-0.53 ± 0.05
c	-0.25 ± 0.01	0.31 ± 0.01	-0.069 ± 0.001
ϕ	0.95 ± 0.01	0.97 ± 0.01	0.92 ± 0.01

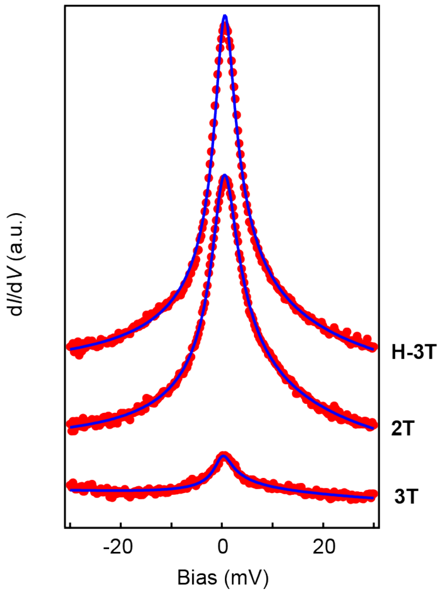

Figure S7 Frota fitting parameters. The solid blue lines show the best fit of 2T, H-3T and 3T ZBRs with the Frota function[2] $-a \cdot Re\left[e^{i\pi\phi}\sqrt{\frac{i\cdot 0.39\Gamma}{eV - x_0 + i\cdot 0.39\Gamma}}\right] + b \cdot V + c$. Where a, b, c parameters represent the amplitude factor, a linear slope of the dI/dV curve and a conductance offset, respectively. ϕ is a phase factor defining the shape of the Kondo resonance, x_0 and Γ are respectively the energy position of the resonance and its half width at half maximum (HWHM). The fitting parameters of each system are listed in the table.

3. Synthetic procedures

Solution synthesis of H-2T

1*H*-phenalene (H-2T) was prepared from commercially available 3-(naphthalen-1-yl)propanoic acid (1) in three steps. The phenalene-core was built up by a Friedel–Crafts acylation. The reduction of 2,3-dihydro-*1H*-phenalen-1-one (2) and a subsequent dehydration of 2,3-dihydro-1*H*-phenalen-1-ol (3) yielded H-2T. The air sensitive compound was purified by column chromatography and subsequent sublimation. Hereafter a detailed description of the synthesis steps of H-2T is reported.

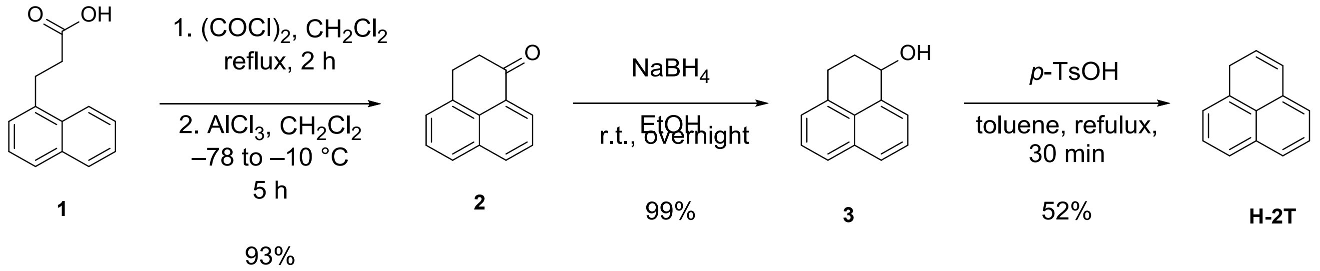

2,3-Dihydro-1*H*-phenalen-1-one (2).[5] The reaction was carried out under inert conditions. A solution of 3-(naphthalen-1-yl)propanoic acid (**1**, 4.50 g, 22.5 mmol) in oxalyl chloride (60 mL) was refluxed for 2 h. After excessive oxalyl chloride was removed under reduced pressure, the crude product was dissolved in CH_2Cl_2 (60 mL) and cooled to –78 °C. $AlCl_3$ (4.5 g, 33.8 mmol) was added to the solution before the reaction mixture was allowed to warm up to –10 °C over a period of 5 h. The reaction mixture was subsequently poured on ice and the organic layer was separated before the aqueous layer was extracted with CH_2Cl_2. The combined organic layers were washed with aq. $NaHCO_3$ (sat.) and were dried over $MgSO_4$. After evaporation of the solvents, the crude product was purified by flash column chromatography (SiO_2, CH_2Cl_2) to afford the desired compound as an off-white solid (3.80 g, 20.9 mmol, 93%). [CAS 518-85-4] **^{1}H NMR (400 MHz, $CDCl_3$, ppm):** δ 8.21 (dd, J = 7.2, 1.3 Hz, 1H), 8.10 (dd, J = 8.2, 1.3 Hz, 1H), 7.81 (dd, J = 7.9, 1.6 Hz, 1H), 7.61 (dd, J = 8.2, 7.2 Hz, 1H), 7.55–7.43 (m, 2H), 3.49–3.40 (m, 2H), 2.99 (dd, J = 7.9, 6.3 Hz, 2H). **^{13}C NMR (101 MHz, $CDCl_3$, ppm):** δ 198.7, 134.2, 133.5, 133.4, 131.8, 130.0, 126.4, 125.8, 125.7, 125.2, 38.7, 28.7. **HRMS (EI) *m/z*:** $[M]^+$ Calcd for $C_{13}H_{10}O$ 182.0726; Found 182.0724.

2,3-Dihydro-1*H*-phenalen-1-ol (3).[5] To a solution of 2,3-dihydro-1*H*-phenalen-1-one (**2**, 300 mg, 1.65 mmol) in EtOH (7 mL) was added $NaBH_4$ (98.7 mg, 1.82 mmol) in one portion. The reaction mixture was stirred at rt overnight before it was quenched with water. The reaction mixture was extracted with CH_2Cl_2, the organic layers were washed with aq. $NaHCO_3$ (sat.), dried over $MgSO_4$ and the solvent was evaporated. The crude product was purified by flash column chromatography (SiO_2, cyclohexane/EtOAc 4:1 v/v) to afford the desired compound as a colorless solid (304 mg, 1.65 mmol, 99%). [CAS 130292-28-3] The obtained ^{1}H-NMR spectrum is in accord with the previously reported data[6]. **^{1}H NMR (400 MHz, $CDCl_3$, ppm):** δ 7.80 (dd, J = 8.2, 1.3 Hz, 1H), 7.72 (dd, J = 8.2, 1.1 Hz, 1H), 7.57 (dt, J = 7.0, 1.2 Hz, 1H), 7.48 (dd, J = 8.2, 7.0 Hz, 1H), 7.42 (dd, J = 8.2, 6.9 Hz, 1H), 7.31 (dq, J = 7.0, 1.3 Hz, 1H), 5.12 (dd, J = 6.6, 3.8 Hz, 1H), 3.40–3.04 (m, 2H), 2.29–2.13 (m, 2H), 1.81 (s, 1H). **^{13}C NMR (75 MHz, $CDCl_3$, ppm):** δ 137.5, 135.2, 133.8, 128.8, 128.2, 126.0, 125.8, 125.7, 124.5, 123.6, 69.5, 31.4, 26.3. **HRMS (EI) *m/z*:** $[M]^+$ Calcd for $C_{13}H_{12}O$ 184.0883, Found 184.0883.

1*H*-Phenalene (H-2T).[5] The reaction and workup were carried out under inert conditions, solvents were deoxygenated. A solution of 2,3-dihydro-1*H*-phenalen-1-ol (**3,** 151 mg, 0.82 mmol) in toluene (5 mL) was refluxed, before a catalytic amount of *p*-TsOH was added. The reaction mixture was refluxed for further 0.5 h. Then, the reaction mixture was cooled to room temperature und concentrated in vacuum. The residing liquid was diluted with petroleum ether and passed through a pad of Florisil with petroleum ether as eluent. The solvent was removed in vacuum and the residing solid was sublimed (~110 °C, $2*10^{-2}$ mbar) to afford the desired compound as a colorless solid (70.3 mg, 0.42 mmol, 52%). CAS [203-80-5] **MP.:** 84–96 °C (Lit. 83–84 °C)[7]. The characterization of 1*H*-phenalene in literature is incomplete, we here report ^{1}H and ^{13}C resonances in CD_2Cl_2 and its high-resolution mass spectrum. **^{1}H NMR (400 MHz, CD_2Cl_2, ppm):** δ 7.55 (dd, J = 8.1, 1.4 Hz, 1H), 7.52 (dd, J = 8.4, 1.1 Hz, 1H), 7.39–7.32 (m, 1H), 7.27 (dd, J = 8.3, 6.9 Hz, 2H), 6.98 (dd, J = 6.9, 1.3 Hz, 1H), 6.60 (dt, J = 9.9, 2.2 Hz, 1H), 6.06 (dt, J = 9.9, 4.1 Hz, 1H), 4.10–4.03 (m, 2H). **^{13}C NMR (101 MHz, CD_2Cl_2, ppm):** δ 134.7, 134.0, 132.4, 129.8, 128.4, 127.8, 127.0, 126.6, 126.5, 125.3, 122.4, 32.4. **HRMS (EI) m/z:** $[M]^+$ Calcd for $C_{13}H_{10}$ 166.0778, Found 166.0766.

4. High-resolution mass spectra and NMR characterization

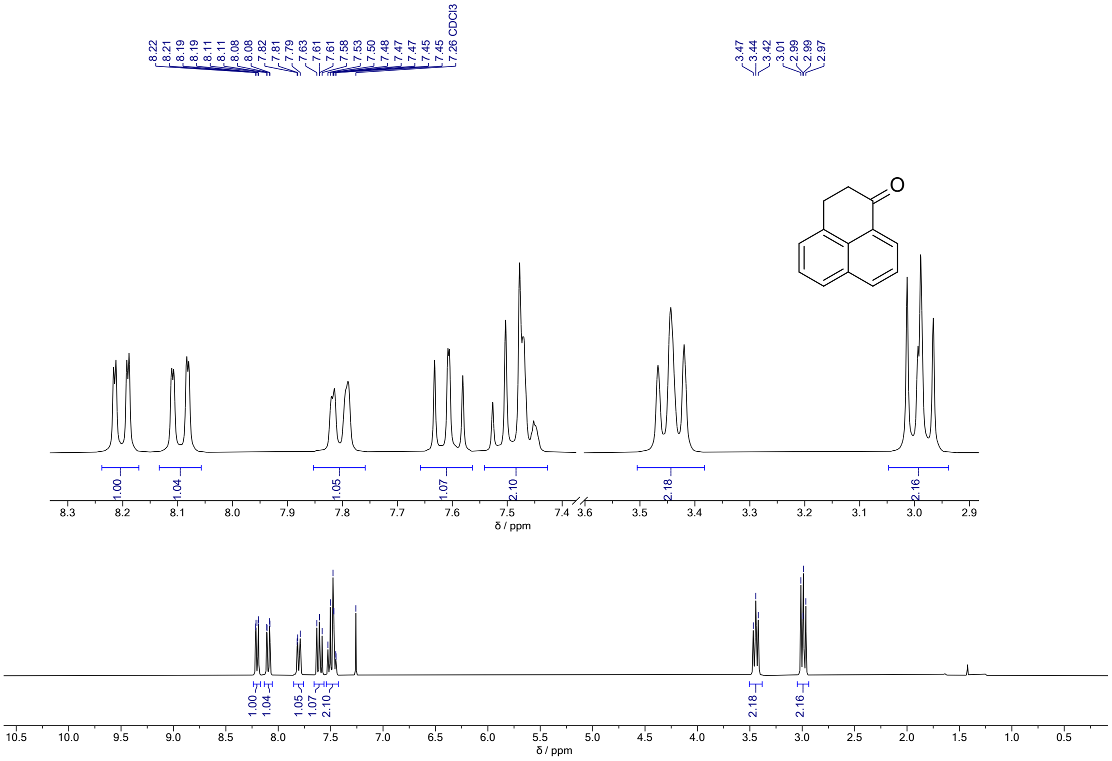

^{13}C NMR / 101 MHz / $CDCl_3$ / 25 °C

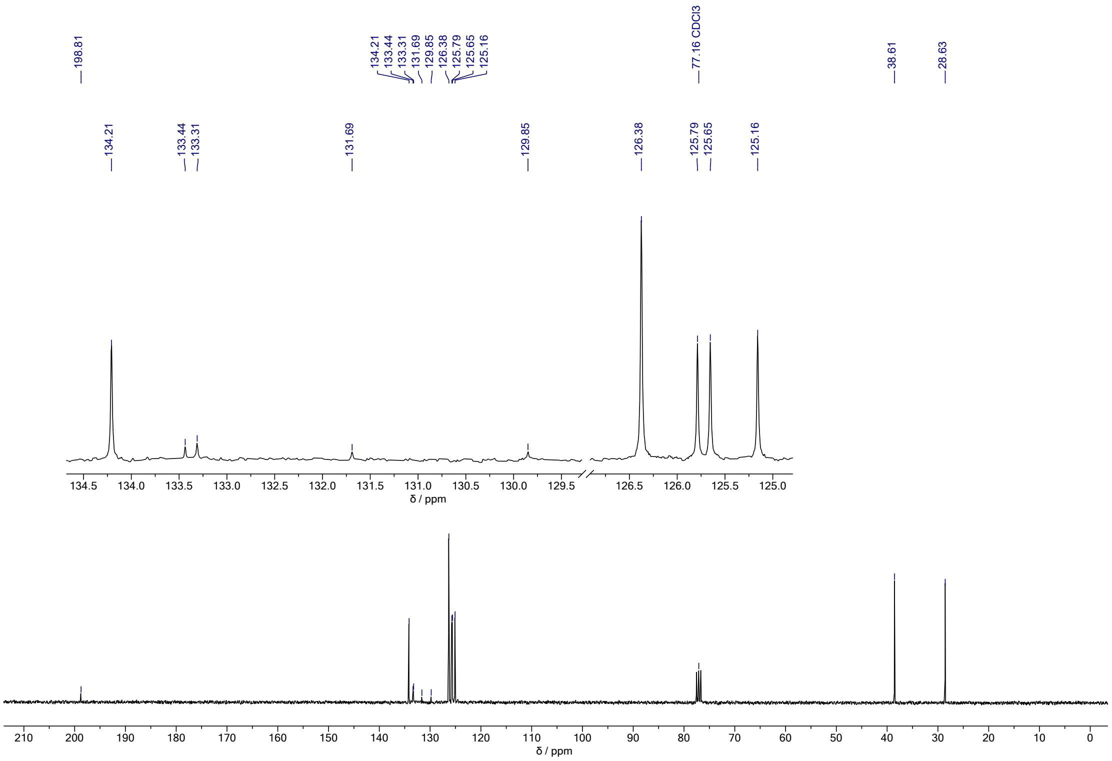

2,3-Dihydro-1*H*-phenalen-1-ol (151) ^{1}H NMR / 400 MHz / $CDCl_3$ / 25 °C

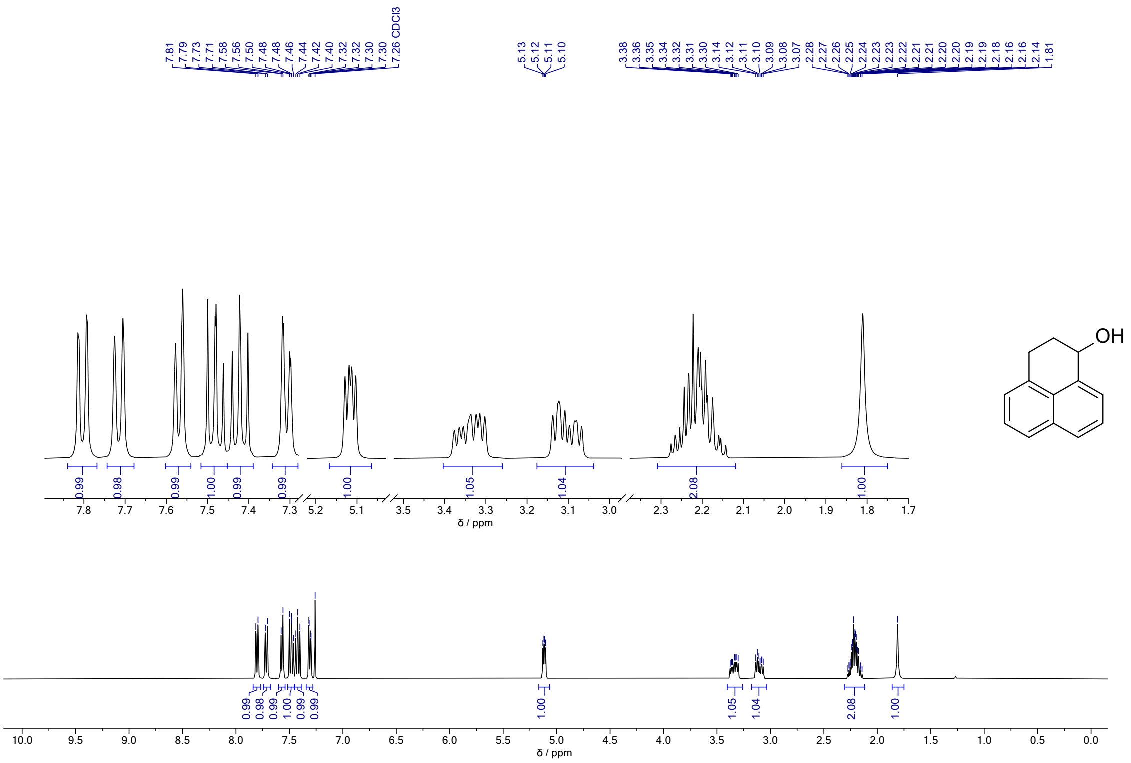

^{13}C NMR / 101 MHz / $CDCl_3$ / 25 °C

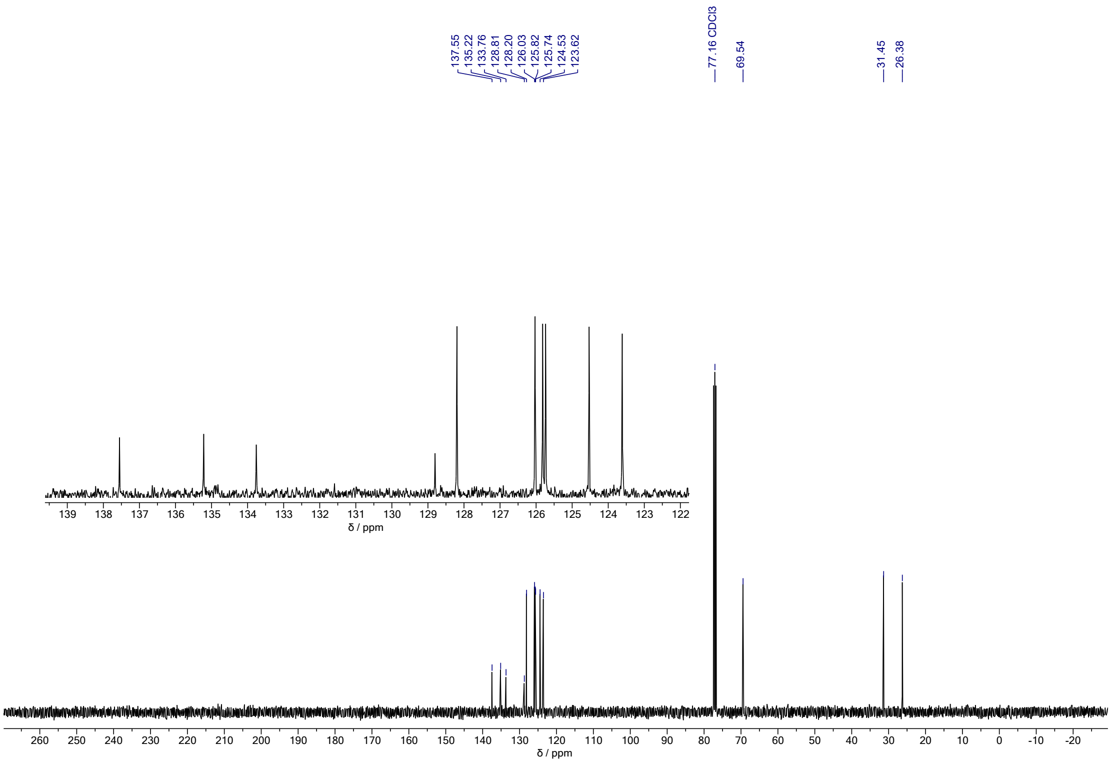

HRMS (EI)

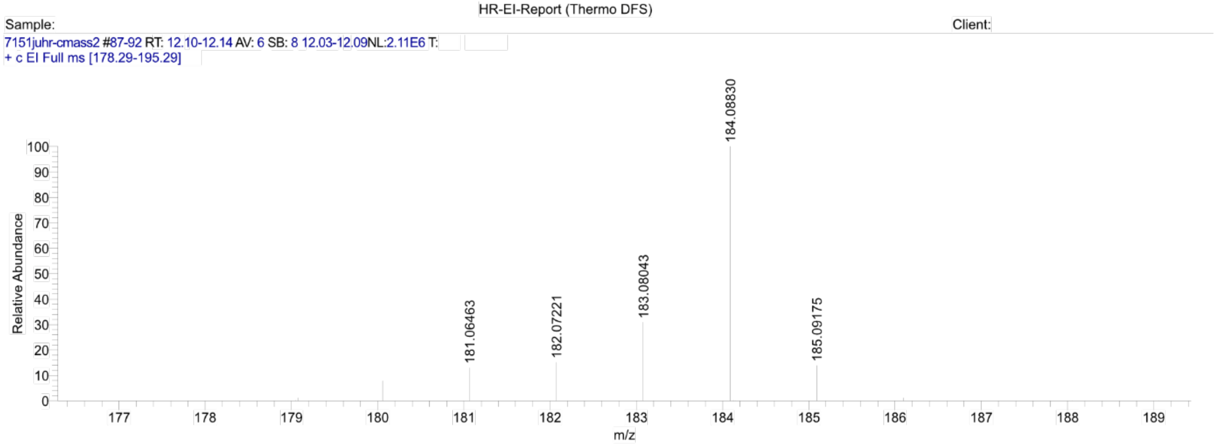

7151juhr-cmass2#87-92 RT: 12.10-12.14 AV: 6 T: + c
EI Full ms [178.29-195.29]
m/z= 180.78581-184.29280

m/z	Intensity	Relative	Theo. Mass	Delta (ppm)	Composition
181.06463	275894.8	13.05	181.06479	-0.88	$C_{13}H_9O$
182.07221	320293.7	15.15	182.07262	-2.23	$C_{13}H_{10}O$
183.08043	652154.2	30.86	183.08044	-0.07	$C_{13}H_{11}O$
184.08830	2113606.0	100.00	184.08827	0.18	$C_{13}H_{12}O$

1*H*-Phenalene (152) ^{1}H NMR / 400 MHz / CD_2Cl_2 / 25 °C

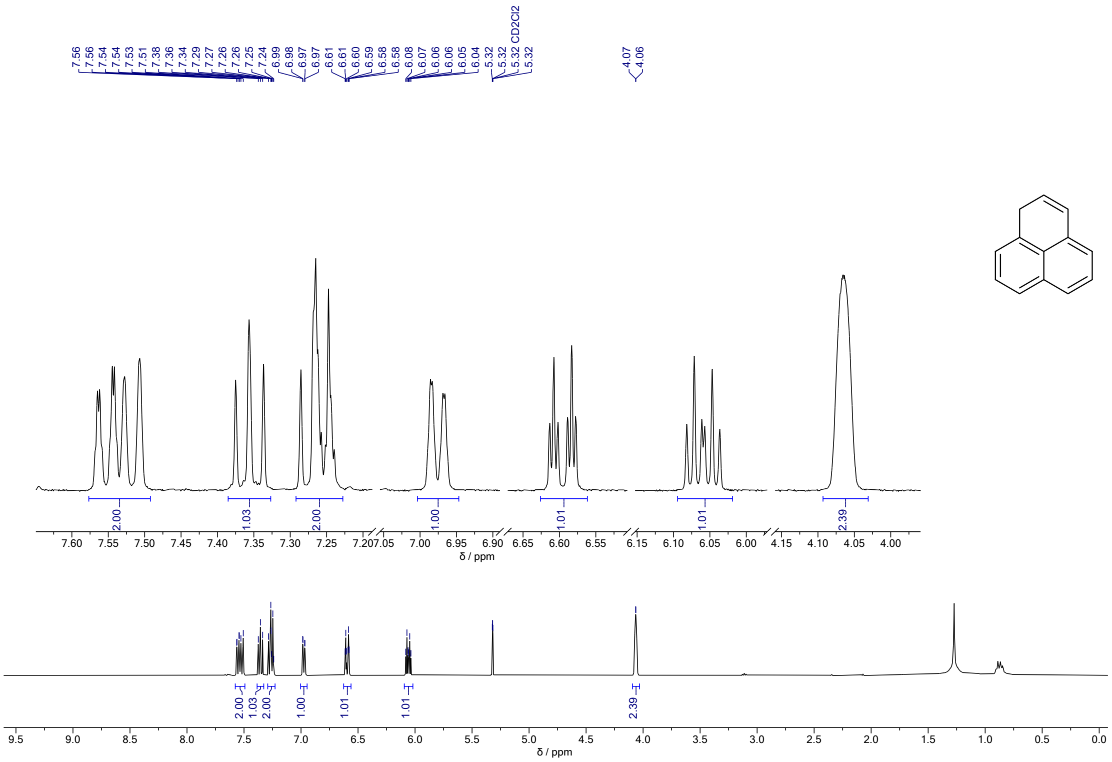

^{13}C NMR / 101 MHz / CD_2Cl_2 / 25 °C

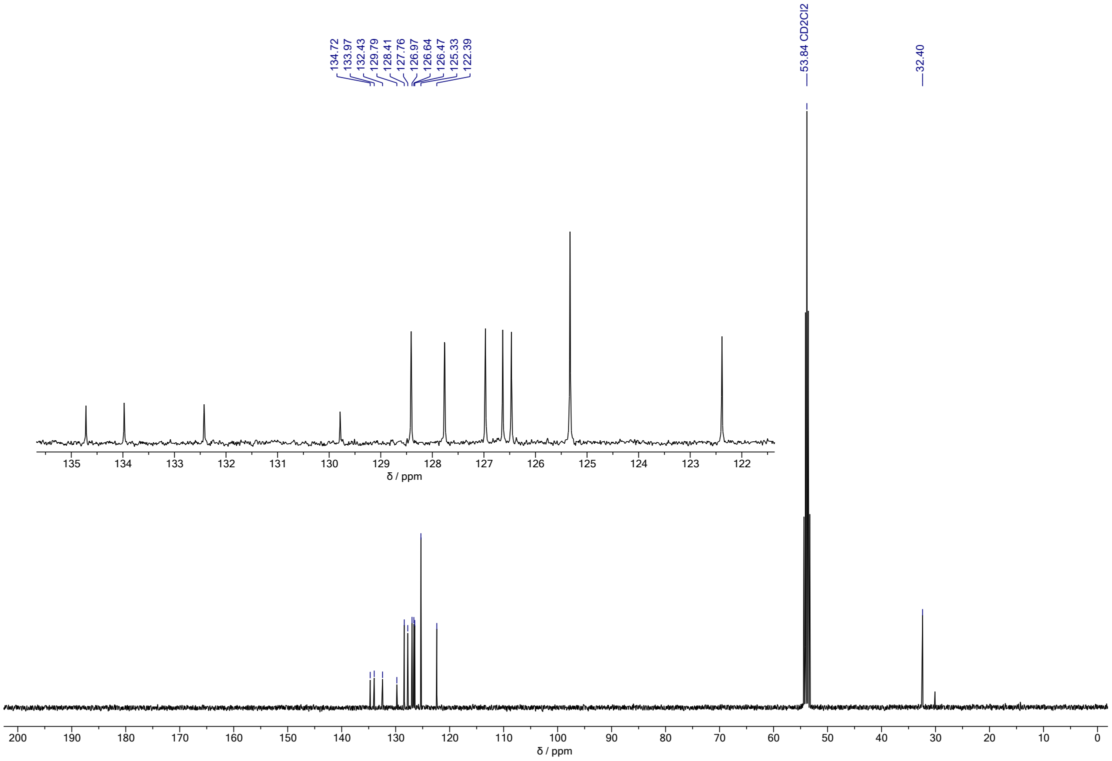

HRMS (EI)

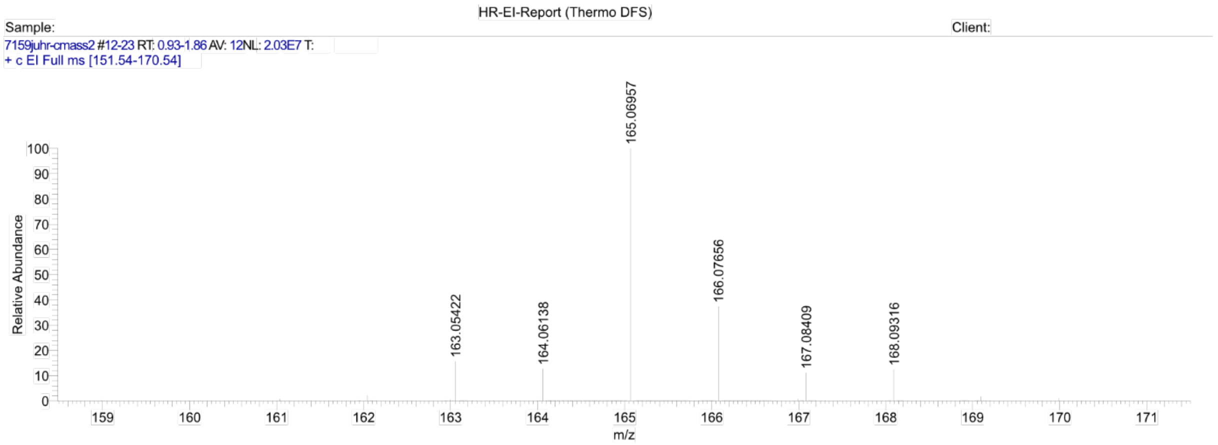

7159juhr-cmass2#12-23 RT: 0.93-1.86 AV: 12 T: +
c EI Full ms [151.54-170.54]
m/z= 162.40806-168.34751

m/z	Intensity	Relative	Theo. Mass	Delta (ppm)	Composition
163.05422	3167051.6	15.64	163.05423	-0.05	$C_{13}H_7$
164.06138	2569084.3	12.69	164.06205	-4.07	$C_{13}H_8$
165.06957	20250922.7	100.00	165.06988	-1.83	$C_{13}H_9$
166.07656	7566182.8	37.36	166.07770	-6.88	$C_{13}H_{10}$
167.08409	2248272.6	11.10	167.08553	-8.60	$C_{13}H_{11}$
168.09316	2509202.2	12.39	168.09335	-1.17	$C_{13}H_{12}$

6. References

(1) Braun, T. MatrixFileReader, 2021.

(2) Warner, B.; El Hallak, F.; Pruser, H.; Sharp, J.; Persson, M.; Fisher, A. J.; Hirjibehedin, C. F. Tunable Magnetoresistance in an Asymmetrically Coupled Single-Molecule Junction. *Nat Nanotechnol* **2015**, *10* (3), 259–263. https://doi.org/10.1038/nnano.2014.326.

(3) Tran, V.-T.; Saint-Martin, J.; Dollfus, P.; Volz, S. Third Nearest Neighbor Parameterized Tight Binding Model for Graphene Nano-Ribbons. *AIP Adv.* **2017**, *7* (7), 075212. https://doi.org/10.1063/1.4994771.

(4) Eimre, K. Eimrek/Tb-Mean-Field-Hubbard: V1.2.0, 2021. https://doi.org/10.5281/zenodo.4708340.

(5) Ravat, P.; Šolomek, T.; Rickhaus, M.; Häussinger, D.; Neuburger, M.; Baumgarten, M.; Juríček, M. Cethrene: A Helically Chiral Biradicaloid Isomer of Heptazethrene. *Angew. Chem. Int. Ed.* **2016**, *55* (3), 1183–1186. https://doi.org/10.1002/anie.201507961.

(6) Stulgies, B.; Pigg, D. P.; Kaszynski, P.; Kudzin, Z. H. 9,9-Dimethyl-8,10-Dioxapentacyclo[5.3.0.02,5.03,5.03,6]Decane and Naphthotetracyclo[5.1.0.01,6.02,7]Oct-3-Ene: New Substituted [1.1.1]Propellanes as Precursors to 1,2,3,4-Tetrafunctionalized Bicyclo[1.1.1]Pentanes. *Tetrahedron* **2005**, *61* (1), 89–95. https://doi.org/10.1016/j.tet.2004.10.057.

(7) Boudjouk, P.; Johnson, P. D. Improved Routes to Phenalene and Phenalanone. Alane, Borane, and Silane Reductions of Phenalenone. *J. Org. Chem.* **1978**, *43* (20), 3979–3980. https://doi.org/10.1021/jo00414a044.
